# Reevaluating the classification of pediatric speech sound disorders: a ground truthing perspective

**DOI:** 10.3389/fnhum.2025.1700505

**Published:** 2025-12-11

**Authors:** Aravind K. Namasivayam, Raymond Kent, Jonathan L. Preston, Ben A. M. Maassen, Christina Hagedorn, Ignatius S. B. Nip, Anita McAllister, Jun Wang, Katherine Hustad, Lucie Ménard, Nilgoun Bahar, Jennifer Golabek Moore, Julia Petrosov, Pascal van Lieshout

**Affiliations:** 1 Oral Dynamics Laboratory, Department of Speech-Language Pathology, University of Toronto, Toronto, ON, Canada; 2 University of Wisconsin-Madison, Madison, WI, United States; 3 Department of Communication Sciences and Disorders, Syracuse University, Syracuse, NY, United States; 4 Center for Language and Cognition Groningen, University of Groningen, Groningen, Netherlands; 5 Linguistics Program, College of Staten Island, City University of New York, Staten Island, NY, United States; 6 School of Speech, Language, and Hearing Sciences, San Diego State University, San Diego, CA, United States; 7 Division of Speech-Language Pathology, CLINTEC, Karolinska Institutet, Stockholm, Sweden; 8 Department of Speech, Language, and Hearing Sciences, Moody College of Communication, The University of Texas at Austin, Austin, TX, United States; 9 Department of Communication Sciences and Disorders, Waisman Center, University of Wisconsin – Madison, Madison, WI, United States; 10 Department of Linguistics, University of Quebec in Montreal, Montreal, QC, Canada; 11 Department of Neurology, Dyslexia Center, UCSF, San Francisco, CA, United States; 12 Brave Wings Therapy, Fairfield, NJ, United States

**Keywords:** speech sound disorders, speech sound disorder classification, perceptual bias, ground truthing, instrumental phonetics, pediatric speech pathology

## Abstract

Pediatric Speech Sound Disorders (SSDs) are conventionally diagnosed using auditory-perceptual assessments, heavily relying on International Phonetic Alphabet (IPA) transcriptions. This approach, while prevalent, is increasingly criticized due to inherent perceptual biases, limited sensitivity to subtle speech motor variations, and insufficient reflection of underlying speech mechanisms. This paper critically re-examines a widely used diagnostic classification system for pediatric SSDs, namely Dodd’s Model of Differential Diagnosis (MDD), emphasizing the limitations of perceptual methods and advocating for instrumental techniques to address significant ground truthing issues. Critical analysis in this paper integrates evidence from perceptual research, instrumental phonetics, and speech motor development studies, highlighting discrepancies between traditional classification methods and modern instrumental data. Findings indicate profound limitations in current auditory-perceptual classification methods, particularly regarding their inability to detect subtle motoric impairments such as jaw sliding, covert motor contrasts, and undifferentiated tongue gestures. Evidence from instrumental studies supports a speech-motor rather than purely cognitive-linguistic basis for many pediatric SSDs, revealing significant inadequacies in current clinical practices. To avoid the narrow interpretation of “motor speech” as referring only to childhood apraxia of speech (CAS) or dysarthria, we explicitly broaden its scope to include a wider range of motoric influences on SSDs. Given these critical ground truthing concerns, the paper proposes adopting instrumental-based methodologies that offer greater precision in identifying underlying motor-based impairments, thereby promoting a more accurate and nuanced understanding of pediatric SSDs. Furthermore, the discussion advocates for adopting a dimensional rather than categorical classification framework, emphasizing gradual developmental trajectories and foundational speech motor skills. Aligning with modern precision medicine principles, the proposed approach aims to refine diagnostic accuracy, improve intervention effectiveness, and ultimately enhance clinical outcomes for children with SSDs.

## 1 Introduction

### 1.1 Statement of problem

Through the millennia, historical epochs reflect humanity’s evolving understanding of the natural world. An era once tethered to the convictions of a flat Earth and a geocentric model of the universe, attributing diseases to “miasma” (“bad air” i.e., pollution), and accepting systematic treatises on sorcery and witchcraft as prevailing truths, have now yielded to modern scientific inquiry. Today, with the advent of modern science, these beliefs and notions have been debunked. This transformative journey underscores the pivotal role of technological advancements and has taught us that without the right tools (i.e., microscope or telescope in the above examples), our ground truthing is incorrect. We define ground truthing as the process of establishing information as real or accurate through direct observation and measurement (i.e., empirical evidence), as opposed to information derived from assumptions or inferred conclusions (e.g., Arza et al., 2019; Gozzi et al., 2024).

The present paper addresses a long-standing ground truthing concern in the study of pediatric speech sound disorders (SSDs), which are estimated to impact nearly one-tenth (8–9%) of young children (National Institute on Deafness and Other Communication Disorders, 2025). The American Speech-Language-Hearing Association (2025) defines speech sound disorders as an umbrella term referring “to any difficulty or combination of difficulties with perception, motor production, or phonological representation of speech sounds and speech segments—including phonotactic rules governing permissible speech sound sequences in a language.” As noted by Kent (2024), this definition mentions both speech sounds and speech segments without defining the terms, explaining how they differ, or discussing why both are needed. The definition encompasses difficulties of perception, motor production, and phonological representation, while missing the essential construct by which speech sound disorders are recognized—production errors. Despite technological advancements of the 21st century, critical diagnostic assessments of pediatric SSDs continue to rely heavily on human senses (primarily auditory-perceptual), the interpretation of which is inherently subjective and impressionistic, to ascertain the nature, etiology, and classification of these disorders. The utilization of auditory-perceptual assessments marks a persistent pattern in the profession, despite inherent limitations of such perceptual judgments (e.g., Kent, 1996; Munson et al., 2010; Richtsmeier, 2010). Ultimately, the reader will be encouraged to critically evaluate the validity of currently widely used schemes for classifying SSD into subtypes like consistent and inconsistent phonological disorders (Cleland et al., 2025; Dodd, 2014). The classification of SSD subtypes, often based solely on surface-level data derived from a listener’s perceptual, impressionistic International Phonetic Alphabet (IPA) transcription, is unlikely to accurately reflect the complex underlying processes occurring within the child’s speech mechanism. This is the crux of the *ground truthing problem*. This paper focuses on (i) elucidating serious ground truthing problems in pediatric SSD diagnostic classification systems and (ii) offering potential alternatives for characterizing these speech issues in children. We believe that, with appropriate instrumental tools and speech tasks, a more precise understanding of the underlying nature of SSDs and their subtypes can be achieved (Cleland et al., 2020; Hagedorn and Namasivayam, 2024; Kent, 1996; Meyer and Munson, 2021; Munson et al., 2010; Namasivayam et al., 2020). This view aligns with frameworks that take a broader approach to describing speech sound errors in children, such as those proposed by Edwards et al. (1999), Munson et al. (2010), and Maassen and Terband (2024).

This paper begins by outlining proximal concerns with the clinical classification of SSDs, including biases in perceptual evaluation, the challenges of clinical subtyping, and the lack of integration of instrumental evidence and contemporary research on speech motor development. It then turns to broader, distal issues—most notably, methodological flaws in data sampling that underlie widely adopted classification systems, with a particular focus on Dodd’s Model of Differential Diagnosis (MDD; Dodd, 2014). While MDD is central to this critique—especially in light of recent accelerated efforts to prematurely establish consensus and standardize MDD-based categorical diagnostic “labels” within policy and service frameworks (e.g., Cleland et al., 2025; Csercsics et al., 2024; Stringer et al., 2024), this paper also challenges any classification system that fails to integrate current knowledge of the motoric, perceptual, linguistic, and social-cognitive foundations of speech and language development (e.g., the Speech Disorders Classification System (SDCS); Shriberg et al., 2010).

The discussion reconsiders SSD classification through a modern instrumental lens, integrating emerging findings on speech motor system development. Finally, the paper offers an alternative perspective on classification, drawing parallels with dimensional versus categorical frameworks in psychiatry. This work aims to initiate a broader dialog and highlight the complexity of characterizing and classifying pediatric SSDs, emphasizing the importance of thoroughly assessing the foundational skills that support speech-sound learning before adopting standardized diagnostic practices. Before we proceed further, it is important to clarify the use of motor-related terminology in this manuscript. The term “motor speech” is often narrowly associated with conditions such as childhood apraxia of speech (CAS) or dysarthria. This restricted interpretation risks obscuring broader motoric contributions to pediatric SSDs, such as adaptive jaw compensation, undifferentiated tongue movements, and covert speech motor contrasts, which may not fit neatly within those clinical categories. Given the semantic baggage inherent in established classification systems, careful definition of motor-related terms is essential to avoid reinforcing reductive interpretations and to ensure that a wider spectrum of motor influences on speech development is recognized.

In this manuscript we distinguish the terms impairment/disorder/deficit from “limited” in the context of speech-motor skills. We use impairment/disorder/deficit to refer to speech motor problems linked to known neurological or medical conditions (e.g., muscular dystrophy, traumatic brain injury, genetic syndromes, childhood dysarthria) and to disordered motor control. By contrast, when motor performance is inadequate but there is no observable anatomical, physiological, or cognitive pathology (as noted in idiopathic SSDs), we refer to limited speech motor skills. Comparable “limitations” in motor ability have been noted in developmental coordination disorder (Dewey and Wilson, 2001), in children with low motor competence (Hands, 2008), and in individuals who stutter (Namasivayam and Van Lieshout, 2011; Van Lieshout et al., 2004). Here, “limitation” denotes reduced performance, but it typically remains within the broad ranges seen in healthy individuals, and resembles movements seen particularly at earlier stages of skill acquisition.

We define speech motor skill as the capacity to learn, through practice, the movements required for efficient task execution and to perform them in a highly organized, largely automatic, adaptable, energy efficient, and goal directed manner (Namasivayam and Van Lieshout, 2011; Van Lieshout et al., 2004). Individuals with limited (speech) motor skills may show: (a) frequent errors with reduced movement consistency or high variability (Ackerman, 2007; Newell, 1991); (b) slow execution and greater reliance on sensory feedback (Atkeson, 1989; Halsband and Lange, 2006); (c) reduced automaticity and vulnerability to interference from concurrent tasks (Poldrack et al., 2005); (d) insufficient functional adaptation to meet task demands (Chow et al., 2007); (e) immature motor patterns and a lack of finely graded, well differentiated movements tailored to the task (Chow et al., 2007; Smits-Engelsman and Van Galen, 1997); (f) difficulty achieving high performance despite extensive practice, or slow development of high performance with practice (Ackerman, 2007); (g) inefficient movements in terms of time and energy, with limited use of the intrinsic dynamics of the neuromusculoskeletal system in a manner appropriate for the task (Chow et al., 2007; Newell, 1991; adapted from Namasivayam and Van Lieshout, 2011).

Within the domain of speech motor skills, strategies can be described as sets of self-organized, optimal solutions that are tuned for stability, physiological effort, and related factors which emerge from dynamic interactions among task goals and multiple constraints. These constraints include task requirements (for example, speech rate and lexical stress), personal factors (for example, neuromuscular and oro-facial characteristics), and environmental conditions (for example, speaking in noise). Task goals refer to appropriate speech targets, which may be specified in gestural terms (Goldstein et al., 2007) and in somatosensory and auditory terms (Guenther, 2006). Such strategies furnish the speaker with a repertoire of optimal actions or parameter settings for controlling muscle force and timing. They should not be confused with deliberate cognitive strategies, such as choosing particular words or phrases to avoid stuttering (Namasivayam and Van Lieshout, 2011). Motor skill levels vary across individuals and fall along a continuum (Smyth, 1992; Van Lieshout et al., 2004). Because the variation is continuous, any cutoff separating limited from high skills is to some extent arbitrary (Keogh et al., 1979; Namasivayam and Van Lieshout, 2011; Van Lieshout et al., 2004).

## 2 Ground truthing problem in pediatric SSD

### 2.1 Perceptual bias and limitations of IPA transcription

We begin with the IPA, which was originally designed for descriptive (not diagnostic) purposes. Over time, however, clinicians began using phonological process labels derived from IPA transcriptions as explanatory constructs rather than mere descriptions (Locke, 1983). Today, widely used diagnostic frameworks continue to rely heavily on auditory-perceptual IPA transcription (Waring and Knight, 2013), assuming these transcriptions are veridical and capable of revealing underlying causes—an assumption that often misguides classification systems (e.g., Dodd, 2014).

#### 2.1.1 Do we trust our ears?

Auditory-perceptual, IPA transcription-based assessments have a long history in pediatric SSDs (Waring and Knight, 2013). Assessment typically involves phonetic transcription of children’s speech, a task rooted in categorical perception: speech-language pathologists (SLPs) assign symbols such as /d/ or /g/ to represent place, manner, and voicing in a child’s productions. However, as Cutler argues in *Native Listening* Cutler (2012), speech perception is exquisitely tuned by native-language experience. This tuning makes everyday communication efficient but can work against fine-grained, segment-by-segment phonetic analyses. Consequently, categorical perception can complicate the interpretation of transcribed error types (e.g., backing: perceived sub stitution of velar /k/ for alveolar /t/ as in “cop” for “top”) and judgments of their consistency. Yet transcribed error types and their consistency remain the predominant basis for differentiating SSDs, including phonological delay, consistent phonological disorder, and inconsistent phonological disorder (e.g., Dodd, 2014).

This approach also presumes that the segments clinicians perceive and transcribe perfectly reflect the segments represented and produced by the child’s speech system (Kent, 1996). That presumption is problematic: perceptual transcriptions capture clinicians’ interpretations rather than exact records of the child’s articulatory events (Kent, 1996). Below are additional, converging lines of evidence indicating that exclusive reliance on transcribed error types to categorize SSD subtypes is ill-advised, despite their widespread use for differential diagnosis in contemporary practice (Cleland et al., 2025; Csercsics et al., 2024).

The most salient argument against using perceptual transcription as the basis of SSD classification is the strong biases inherent to our auditory-perceptual system which preclude perceptual transcription from reflecting objective reality. Research has demonstrated that listeners often “fill in” sounds and phoneme features that are absent in the acoustic signal through a process known as phoneme restoration (Warren and Sherman, 1974). Furthermore, our perception is shaped by top-down cognitive influences and priming effects, leading us to hear what we expect rather than what is actually present in the speech signal (e.g., Davis and Johnsrude, 2007). For example, perception is influenced primarily by listeners’ own native language backgrounds and the respective phonologies of those language varieties. A given production will, therefore, be perceived by listeners with distinct linguistic profiles in distinct ways. Listeners also subconsciously integrate multisensory inputs, which can distort their perception of reality—examples include the McGurk effect (Alsius et al., 2018) and aero-tactile integration (Gick and Derrick, 2009).

While going into depth on each of the factors that limit the validity and reliability of perceptual judgments of speech and voice disorders is beyond the scope of this article, we refer the reader to Kent (1996), which discusses these in detail. The seriousness of the impact of perceptual bias on SSD classification is evident in the following statement: “It can be very easy to confuse level of description with level of explanation. Just because phonemic errors are easy to detect does not mean that the disorder process necessarily operates exclusively or primarily at a phonemic level of speech organization” (Kent, 1996, p. 13). This statement prompts us to question the validity of the classification models anchored in the descriptive–linguistic approach, where SSD subtype classification is primarily based on error patterns observed and transcribed (e.g., Dodd, 2005, 2014; Grunwell, 1997; Kamhi, 1992).

#### 2.1.2 IPA transcription forces phonemic categories on errors that exist along a continuum

The classification of SSD subtypes through perceptual error categorization is further complicated by the reliance on phonemic transcription using the IPA, which imposes discrete phonemic categories on errors that likely exist along a phonetic continuum. A recent study by Meyer and Munson (2021) examined how individuals with varying levels of clinical experience—including experienced and less experienced SLPs as well as non-clinicians—perceived children’s speech production using continuous rating scales. Participants rated the place of articulation for children’s word-initial productions of /θ/, /s/, /ʃ/, /d/, /ɡ/, /t/, and /k/ on a nine-point scale. The stimuli included productions verified instrumentally as intermediate sounds (e.g., a /t/ that was neither fully /t/-like nor fully /k/-like). Surprisingly, less experienced SLPs and non-clinicians provided intermediate ratings for these ambiguous productions, whereas more experienced SLPs were more likely to categorize them at the scale’s endpoints, labeling them strictly as either /k/ or /t/. This suggests that clinical experience and training may, paradoxically, reinforce categorical perception rather than foster sensitivity to graded phonetic variation. If SLPs are trained to categorize phonemes rigidly, they may overlook subtle but meaningful phonetic changes in children’s speech.

This forced categorization of phones into phonemes has significant implications. Broad IPA transcription, commonly used in standardized articulation and phonological assessments [e.g., Diagnostic Evaluation of Articulation and Phonology (DEAP; Dodd et al., 2002), Goldman-Fristoe Test of Articulation 3rd edition (GFTA-3; Goldman and Fristoe, 2015)] and outcome measures such as percent consonants correct, captures linguistic variation but fails to capture the gradual nature of speech development and therapy progress. No motor skill develops instantaneously from nonexistent to mature, yet speech production is often expected to shift abruptly from incorrect to correct (Munson et al., 2010). Research shows that during speech sound acquisition, children may first exhibit subtle sub-phonemic variations that are not perceptible to listeners, making them insufficient to merit a change in traditional IPA notation (Munson et al., 2010; Tyler et al., 1990).

In fact, there is evidence suggesting that non-categorical gradient speech motor change is the norm, not the exception, in both typical and disordered speech development (Hewlett and Waters, 2004; Munson et al., 2010). This is particularly evident in children with SSD, where the acquisition of correct contrasts, such as the apparent shift in velar fronting (e.g., /t/ for /k/), does not occur discretely but through incremental articulatory changes toward correct targets (Cleland and Scobbie, 2021). Speech sound errors by these children may include productions that are intermediate between velar and alveolar regions, where articulatory contact occurs between the velum (for /k/) and alveolar ridge (for /t/) or, in some cases, involves double articulation (involving contact in both places) (Bartle-Meyer et al., 2009; Edwards et al., 1997; Gibbon, 1999; Hagedorn et al., 2017; Hardcastle et al., 1987). Such intermediate productions have also been observed during acquisition of other contrasts such as the /r/−/w/ contrast (McAllister Byun et al., 2016), /s/−/θ/ contrast (Baum and McNutt, 1990), /s/−/ʃ/ contrast (Li et al., 2009), and voiced and voiceless stops (Hitchcock and Koenig, 2013; Macken and Barton, 1980), among others (Munson et al., 2010). These findings align with pioneering work by Mowrey and MacKay (1990), who used acoustic and electromyographic (EMG) analyses to demonstrate that what were previously considered phonemic or segmental errors in adult speech were, in fact, gradual, subphonemic articulatory shifts. Their study highlighted that perceptual transcription forces categorical labels onto errors that emerge along a continuum, raising fundamental concerns about the reliability of traditional phonological classifications. Mowrey and MacKay (1990), in fact, question the validity and reliability of virtually the entire literature pertaining to speech errors in their statement “the problem of error characterization is so pervasive and its effect so great as to render the significance of traditionally collected data corpora questionable” (p. 1311). Frisch and Wright (2002) reinforce this conclusion by stating that their findings “support the claims of a growing number of researchers that transcription is inadequate for complete error coding, as transcription makes incorrect assumptions about the wholly categorical and abstract nature of the data” (p. 159). Expertise is not sufficient to ensure reliability of transcription. Kerswill and Wright (1990) speak to this issue, noting that “there are intervening factors of a psycho-acoustic nature that impinge on a phonetician’s transcription, thus affecting validity; and there is considerable inconsistency, both between phoneticians and between a single phonetician’s different attempts at transcribing the same token” (p. 255).

Additionally, articulatory evidence from adult studies suggests that many speech errors traditionally categorized as phonological substitutions or deletions may instead arise from the co-production of unintended or intrusive gestures, which help maintain dynamic stability in the speech production system (Goldstein et al., 2007; Hagedorn et al., 2017; Pouplier, 2007, 2008; Pouplier and Goldstein, 2005; Slis and Van Lieshout, 2016a,b). This was demonstrated in a study by Goldstein et al. (2007), where participants produced bisyllabic sequences (e.g., “cop top”) at varying speech rates. Rather than true segmental substitutions, both intended and unintended articulatory gestures were simultaneously produced, a pattern rarely captured by traditional transcription methods (Dell et al., 2000). At faster speech rates, additional tongue tip gestures appeared during /k/ in “cop” and tongue dorsum gestures during /t/ in “top,” leading to a phonotactically illicit /kt/ overlap. This phenomenon aligns with dynamical systems theory: as rate increases, coordination reorganizes from a relatively unstable 1:2 coupling—a given onset gesture (tongue tip for /t/ or tongue dorsum for /k/) occurs once across the two-syllable sequence, while the coda /p/ occurs twice—to a more stable 1:1 coupling, achieved when intrusive, non-target gestures appear (tongue tip during /k/, tongue dorsum during /t/). With these intrusions, each onset gesture now occurs in both syllables, matching the two occurrences of /p/ and resulting in illicit /kt/ overlap (Pouplier, 2008; Slis and Van Lieshout, 2016a,b). Importantly, these gestural intrusions often go undetected by listeners, as only large-magnitude intrusions are perceptible and typically transcribed as segmental substitution errors (Mowrey and MacKay, 1990; Pouplier and Goldstein, 2005). This challenges the assumption that errors result solely from phonological misrepresentation, suggesting many speech errors emerge from biomechanical instability and constraints on motor coordination.

#### 2.1.3 IPA transcription is insufficient for reliably classifying SSD subtypes

While IPA transcription is an accessible tool clinically, a major concern with auditory-perceptual IPA transcription is its limited intra- and inter-rater reliability. Shriberg and Lof (1991) warned that phonetic transcription should be approached with caution due to variability in agreement among—and within—transcribers. Their study reported intra- and inter-rater reliability ranging from approximately 60–90%, with broad transcription yielding the highest agreement at 93%, followed by narrow transcription at 74%, while diacritic use exhibited the lowest agreement at 33%. Notably, these figures represent raw percentage agreement and do not account for chance agreement (which would likely reduce reliability further if corrected using Kappa statistics). Even among trained SLPs, narrow phonetic transcription remains highly inconsistent. This may explain why fewer than 1% of SLPs surveyed by Knight et al. (2018) reported using narrow transcription in clinical practice, despite this level of detail being critical for error subtyping and intervention-related progress monitoring (Meyer and Munson, 2021). Researchers have advocated instrument-assisted transcription to improve segmental and suprasegmental reliability (Ball, 2021; Cleland et al., 2020; Kent, 1996; Kreiman et al., 1994; Maxwell and Weismer, 1982; McMurray, 2022; McNeil and Kent, 1990; Meyer and Munson, 2021; Mowrey and MacKay, 1990; Shriberg and Lof, 1991; Terband et al., 2019).

#### 2.1.4 Ratings of global properties such as intelligibility and severity of disorder lack requisite reliability and validity

Often included in the clinical assessment of children’s speech are ratings of the global properties of an utterance, such as intelligibility and severity of disorder. These measures are inherently multidimensional. Therefore, such ratings cannot singularly index speech competence, as they are influenced by a combination of fine-grained levels, including words, phonemes, or subphonemes. The combination of data from different levels can produce a multi-granular analysis in which global properties are correlated with, or interpreted by, data at finer levels. Global properties have value as an overall estimate of communication effectiveness and are relevant to the activities and participation categories in the ICF-2 model (Hustad, 2012). However, studies point to serious deficiencies in the reliability and validity of clinician judgments of these global properties (Allison et al., 2025; Barreto and Ortiz, 2008; Flipsen et al., 2005; Hustad et al., 2015; Moser et al., 2025; Natzke et al., 2020; Sherman and Morrison, 1955). In one of the most recent studies, Allison et al. (2025) reached the sobering conclusion that clinicians’ subjective intelligibility estimates of children’s speech are not sufficiently accurate or reliable. Clinicians should take note of serious questions that have been raised on the reliability and validity of commonly used auditory-perceptual assessments of children’s speech at most levels of analysis. The deficiencies reduce confidence in the use of these ratings to identify subtle impairments, to monitor changes in speech production associated with intervention or exacerbation of the disorder, or to convey results to other parties. This is not to imply that perceptual judgments should be abandoned, but rather to assert that efforts should be made to enhance these methods or supplement them with other methods, as considered later in this paper.

### 2.2 Instrumental evidence challenging perceptual-based classifications

#### 2.2.1 Covert contrast and perceptual neutralization in children with SSDs

Instrumental studies on speech errors in children with idiopathic SSDs have identified covert contrasts in their speech production (Richtsmeier, 2010; Roxburgh et al., 2022). A covert contrast refers to subtle articulatory or acoustic distinctions between speech sounds that are not perceptible to listeners, a phenomenon known as perceptual neutralization. For instance, a child whose productions of /s/ and /ʃ/ are consistently transcribed as [s] may still produce measurable acoustic differences between the two, indicating an underlying awareness that they are distinct phonemes. Such “covert contrast” (Macken and Barton, 1980) will be hidden from listener perception in the absence of instrumentation capable of detecting these differences (Meyer and Munson, 2021). Since IPA transcription is, at best, only as accurate as the perception of the listener, and such contrasts are not detected perceptually, perception based IPA fails to capture the full range of a child’s true linguistic-phonological knowledge (Meyer and Munson, 2021; Scobbie et al., 2000).

These covert contrasts, first identified in acoustic-spectrographic analyses in the 1970s (Kornfeld, 1971; Kornfeld and Goehl, 1974), highlight that adults do not always perceive distinctions that children make (Kornfeld, 1971, p. 462). The gradual emergence of speech sounds via covert contrasts in children was first documented by Macken and Barton (1980) in their study of voicing contrasts in stop consonants. They found that children went through a transitional phase in which voiced stops were perceived instead of voiceless ones, despite acoustic analysis showing longer voice onset times (VOTs) for such voiceless stops as compared to voiced stops. However, because these VOT values still fall within the adult range of target voiced stops in English, listeners classified them as phoneme substitution errors (Macken and Barton, 1980; Munson et al., 2010). Over the years of research that followed, covert contrasts in children’s speech have been observed for a variety of place (e.g., stops), manner (e.g., fricatives), and voicing contrasts (Bressmann et al., 2011; Hitchcock and Koenig, 2013; Li et al., 2009; Macken and Barton, 1980). Berti (2010) reported that for two groups of children (those in the acquisition process of the phonological contrast and those with phonological disorder), the majority of perceptually judged substitution errors were determined by acoustic methods to be covert contrasts.

Empirical data in support for covert contrasts not only come from acoustic analysis (McAllister Byun et al., 2016; Tyler et al., 1990) but also from studies using varied instrumentation including electropalatography (EPG) studies in children and adults with speech impairments (Gibbon, 1990, 1999; Gibbon and Crampin, 2001; Gibbon and Lee, 2017) and ultrasound tongue data from children with velar fronting errors (e.g., Cleland et al., 2017; Cleland and Scobbie, 2021; McAllister Byun et al., 2016) and in cleft lip and palate speech (Roxburgh et al., 2022). In the EPG studies by Gibbon and colleagues (Gibbon, 1990, 1999; Gibbon and Crampin, 2001; Gibbon and Lee, 2017), the widespread occurrence of “undifferentiated lingual gestures” was reported in children with phonological disorders. These gestures are characterized by minimal differentiation among the tongue tip, tongue body, tongue dorsum, and lateral margins. On EPG, they exhibit midsagittal contact extending from the palato-velar to alveolar regions, indicating broad tongue elevation rather than precise, region-specific control (Gibbon, 1999; Namasivayam et al., 2020). The perception of place of articulation in an undifferentiated gesture is influenced by changes in tongue-palate contact during closure, a phenomenon known as articulatory drift (Gibbon and Wood, 2002). For instance, closure may begin in the velar region, extend across the palate, and be released in the coronal or anterior region, or vice versa. As a result, undifferentiated lingual gestures can give rise to the perception of velar fronting or coronal backing. The final tongue-palate contact location and the direction of articulatory drift determine the perceived place of articulation (Gibbon and Wood, 2002). Studies show that while children produce distinct lingual movements differentiating phonological contrasts, listeners may still perceive these contrasts as neutralized or substitution errors (Gibbon, 1999; Namasivayam et al., 2020). The question then arises as to whether phonetic properties of the speaker or categorical perception of the listener should be considered the ground truth.

Critics often highlight the controversy surrounding covert contrast, noting that not all children exhibit these subtle distinctions during development. However, this variability is likely attributable to the diverse ways in which phonetic contrasts can be produced (Cleland et al., 2017, 2020; Meyer and Munson, 2021; Munson et al., 2010; Scobbie et al., 2000). Studies that report no evidence of covert contrast may have examined only a limited set of parameters, potentially overlooking distinctions present in unmeasured domains (McAllister Byun et al., 2016; Munson et al., 2010). Earlier research, for instance, primarily relied on a narrow range of acoustic measures, which may have failed to capture the full range of covert articulatory differences (McAllister Byun et al., 2016; Munson et al., 2010). Overall, the findings suggest that the production of covert contrast is highly prevalent and may represent the norm rather than the exception (see McAllister Byun et al., 2016; Munson et al., 2010 for further discussion).

An important practical question arises at this point: If clinicians cannot perceive the subtle contrasts a child produces, is the presence of such contrasts relevant, given that the child will receive substantial intervention nonetheless? This is where the principles of precision medicine become critical. A clear understanding of the underlying speech production mechanisms not only informs the selection of appropriate intervention strategies and dosage but also holds predictive value. Across multiple studies, children who already show productive knowledge of the target contrast, evidenced acoustically as covert contrast in which productions are distinct despite identical transcription, advance more quickly in treatment than peers without such knowledge (Forrest et al., 1990; Gibbon et al., 1993; Meyer and Munson, 2021; Tyler et al., 1990, 1993). Furthermore, Forrest et al. (1990) found that productive knowledge of the alveolar-velar contrast, as evidenced acoustically, foreshadowed acquisition of that contrast even without direct treatment. Hence, for children who already show such covert contrasts, careful monitoring or perhaps lower-intensity intervention may be appropriate. Thus, if covert contrasts are a natural and frequent stage in speech development, then perhaps children in this stage should be reinforced for their emergence. However, such reinforcement is impossible if the clinician is unaware of the emerging behavior. Overall, accurate identification of subphonemic, gradient patterns in children’s speech production is often clinically valuable and can inform assessment, prognosis, and treatment planning.

#### 2.2.2 Instrumental evidence supports speech-motor/phonetic explanations

This brings us to the next critical point: Is there any experimental evidence, specifically physiological or instrumental data on children with idiopathic SSDs, confirming that the level of description (i.e., error types like velar fronting and backing) aligns primarily with a perceptually based (higher-order) phonological/phonemic level of explanation?

Research in instrumental phonetics has been critical to both dissecting typical trajectories (e.g., Barbier et al., 2020; Noiray et al., 2018; Scobbie et al., 2000; Zharkova et al., 2011) and studying disordered speech populations (Cleland and Scobbie, 2021; Fletcher, 1992; Kornfeld, 1971; Kornfeld and Goehl, 1974; Lundeborg et al., 2015; Macken and Barton, 1980; McAllister Byun et al., 2016; Ohala, 1974; Smith, 1979; Weismer, 1984). Over the past five decades, numerous independent studies utilizing methodologies such as acoustic analysis, electropalatography (EPG), and ultrasound have consistently provided evidence favoring a phonetic/motor rather than a (higher-order) phonological or phonemic explanation of these errors in children with idiopathic SSDs (e.g., Gibbon, 1999; McAllister Byun et al., 2016; Meyer and Munson, 2021; Munson et al., 2010; Roxburgh et al., 2022; see Namasivayam et al., 2020 for an overview on this topic).

Electropalatography (EPG) alone reveals that 71% of children (12 of 17 children aged 4–12 years; Gibbon, 1999) diagnosed with articulation and phonological disorders exhibit undifferentiated lingual gestures. However, this likely underestimates the prevalence of underlying speech-motor skill limitations; see Section 4.3, “Is it a Difference or a Disorder? Speech errors as adaptive and compensatory strategies,” for further discussion. With additional instrumentation such as ultrasound to measure tongue shape differences (Kabakoff et al., 2021; McAllister Byun et al., 2016), kinematic data of inter-articulator coordination (Goldstein et al., 2007; Hagedorn et al., 2017; Namasivayam et al., 2020) and specific acoustic measures (McAllister Byun et al., 2016; Munson et al., 2010), we may see an even greater proportion of these cases ascribed to involve “phonological” delay/disorder to in fact have origins related to phonetic / speech motor limitations (Hagedorn and Namasivayam, 2024; Namasivayam et al., 2020). For an in-depth explanation of how speech motor limitations on inter- and intra-gestural specification and coordination can give rise to SSDs, the reader is directed to Namasivayam et al. (2020) and Hagedorn and Namasivayam (2024).

At this stage, readers might pause to consider that, in the literature (e.g., Broomfield and Dodd, 2005; Dodd et al., 2002), children reported to have phonological delays or disorders are often screened for oro-motor control difficulties and exhibit none. Is it plausible that these children who exhibit no oro-motor control difficulties (as assessed in the literature) indeed have phonetic or speech motor limitations. Here, it is crucial to examine precisely what the oro-motor activities reported in the literature assess. The Oro-Motor Test in the DEAP assesses performance in three tasks: diadochokinesis for the phrase “pat-a-cake”; isolated nonspeech movements, such as moving the tongue from side to side; and sequence movement for nonspeech behaviors such as kiss and cough. Even if movements being classified as speech versus nonspeech are considered to fall on a continuum rather than a dichotomy (a topic of strong debate), the oro-motor tasks assessed by the DEAP fall mainly to the nonspeech side. Except for the DDK task, they do not require actual speech movements and therefore are not valid indicators of faulty speech motor control. Closely matched speech and nonspeech tasks may share activation of similar or overlapping neural networks, but they have been demonstrated to differ in the dynamics of activation (Bose and Van Lieshout, 2012; Lancheros et al., 2020). Moreover, the DEAP (Dodd, 2005; Dodd et al., 2002) has serious limitations in identifying subtle speech motor difficulties such as jaw sliding, adaptive jaw compensation, undifferentiated tongue movements, covert speech motor contrasts, and other developmental speech motor challenges—that may manifest as apparent “phonological” errors (Gibbon, 1999; Mogren et al., 2022; Namasivayam et al., 2020, 2025; Terband et al., 2013). In fact, a recent study demonstrated that phonological error patterns identified by the DEAP (e.g., cluster reduction, final consonant deletion, stopping, gliding, and atypical errors) in preschool-aged children with SSDs were systematically associated with limitations in jaw, labial-facial, and lingual control (Namasivayam et al., 2025; corroborating results from Edwards et al., 1999). It is unrealistic to expect a brief oro-motor screen or a standard oral-mechanism examination to detect these nuances. This limitation reflects the historical context of the DEAP’s development: at the time, linguistic-phonological accounts predominated and empirical understanding of speech-motor development was still emerging. We elaborate on these issues in later sections. Throughout this paper, we use “motor speech” in a broad sense to include motoric contributions to SSD that extend beyond the canonical diagnoses of CAS and dysarthria. We recognize that entrenched classification labels carry semantic baggage that can obscure such contributions.

#### 2.2.3 Evidence from development of speech motor control

The findings presented thus far call into question the reliability of age-of-acquisition norms derived from IPA transcriptions. Yet, the field continues to rely heavily on models of phonological development—particularly involving expected ages of process suppression (Stampe, 1969), age-based error pattern references (e.g., Bowen, 2023, p. 152), and normative acquisition charts based on transcription data (e.g., McLeod and Crowe, 2018). These frameworks often represent the primary “developmental” tools emphasized in SLP training for both assessment and intervention planning. Phonological process development describes typical error patterns children use as they acquire adult-like speech, which gradually disappear (i.e., are “suppressed”) as their speech matures (Stampe, 1969). A common example is stopping, where a child substitutes fricatives like /s/ with stop consonants like /t/, producing “tun” instead of “sun.” Typically, this pattern disappears by approximately age 3 to 4.5 years (DEAP; Dodd et al., 2002). Current clinical training emphasizes the type and age of suppression of these phonological error patterns to distinguish between typical speech development and disorders requiring intervention. However, clinical academic programs have yet to integrate into student clinicians’ training how speech motor development interacts with these domains, despite its significant impact on intelligibility (Namasivayam et al., 2013, 2020, 2025). Decades of research on speech motor development exist, but clinical translation has been slow. A detailed timeline synthesizing speech motor control research and observational data was recently published (see Figure 1 in Namasivayam et al., 2020). Below, we summarize key aspects based on studies of English-speaking children, with further discussion in Namasivayam et al. (2020) on their relationship to speech motor control theories and SSD.

Speech motor development follows a structured progression, where different articulatory components mature at varying rates. In infancy, mandibular movements are basic and primarily limited to simple opening and closing actions due to limited fine force control (e.g., Davis and MacNeilage, 1995; Green et al., 2000, 2002; Kent, 1992; Locke, 1983; Namasivayam et al., 2020; Nip et al., 2009; but see Diepstra et al., 2017; Giulivi et al., 2011). In the first year of life, there is limited lip interaction with the mandible and limited tongue elevation from the mandible (Buhr, 1980; Kent, 1992; Otomo and Stoel-Gammon, 1992). Voicing contrasts emerge as the coordination between the laryngeal and oral articulatory structures (e.g., mandible) are refined around 2 years of age (Green et al., 2002; Grigos et al., 2005; Yu et al., 2014). At that time, children exhibit strong interlip coupling, which later (2–3 years) differentiates to allow independent control of the upper and lower lips, facilitating the production of labiodental fricatives (/f/ and /v/; Green et al., 2000, 2002; Green and Nip, 2010; Stoel-Gammon, 1985; Nip et al., 2009). Between ages two and six, lip movements become increasingly refined (Noiray et al., 2010), and by 3 years, tongue movements increasingly gain independence from the jaw, supporting more precise anterior–posterior lingual actions (Donegan, 2013; Kent, 1992; Otomo and Stoel-Gammon, 1992; Smit et al., 1990; Wellman et al., 1931). At approximately 3–5 years of age, children develop improved tongue-jaw coordination, essential for producing complex speech sounds (Kent, 1992; McLeod and Crowe, 2018). The tongue, a hydrostatic organ, requires both maturation and linguistic experience for finer articulatory coordination (Barbier et al., 2020; Green and Wang, 2003; Kent, 1992; Nittrouer, 1993; Noiray et al., 2013). Thus, sounds such as rhotacized vowels and complex fricatives emerge as the tongue’s subcomponents gain more independence. Speech motor variability continues to evolve, stabilizing between 7 and 12 years of age as coordination between the lips, jaw, and tongue becomes more consistent and efficient (Cheng et al., 2007; Nittrouer, 1993; Nittrouer et al., 1996, 2005; Noiray et al., 2018, 2019a; Smith and Zelaznik, 2004; Zharkova et al., 2011; Zharkova, 2018). During that period, speech motor control interacts with other language-related developments such as vocabulary growth and phonological awareness (Noiray et al., 2019a,b) and reading development (Popescu and Noiray, 2022).

In summary, oral articulatory development follows a structured progression or timeline. Lip–mandible coordination matures earlier than tongue–mandible coordination or the independent movement of tongue subcomponents (Cheng et al., 2007; Terband et al., 2009). Speech motor control develops hierarchically, sequentially, non uniformly, interactively, and over an extended period (Smith and Zelaznik, 2004; Whiteside et al., 2003), significantly impacting speech sound accuracy. To simplify this dynamic process for clinical use and understanding, the development of speech motor control in typically developing English speaking children can be outlined as follows (Namasivayam et al., 2020; see Table 1).

**Table 1:** Speech motor control development in typically developing children (simplified from Namasivayam et al., 2020 for clinical use).

Type of synergy (differentiation)	Approximate age (years)	Supported action
Jaw supported Jaw maturation	0–1.5 years	Speech-related productions are jaw-supported with minimal independent tongue/lip contributions (e.g., low/back unrounded vowels, jaw-assisted bilabials)
Oral – Larynx	1.5–2 years	Voicing onset time control
Upper-lip – lower-lip Lower-lip – jaw	2.5 years	Bilabial productions in high jaw context (e.g., high vowel context – /bi/, /bu/) Independent lower lip movement (e.g., labio-dental consonant: /f/)
Tongue – Jaw	3 years	Lingual transitions in anterior–posterior dimension (e.g., diphthongs: /aʊ/, /ɔɪ/, and /aɪ/) Independent tongue tip/blade elevation (e.g., /t, d, n/) and later supporting grooved /s/, /z/ and laterals/rhotics /l/, /ɹ/
Within tongue (tip-mid-back)	3–5 years	Complex tongue shapes (e.g., required for /ɹ/, /s/ and /l/ consonants)

In a typically developing child under 3 years old, we expect limited independent tongue-tip elevation. Consequently, alveolar consonants may be produced with jaw-supported tongue elevation (jaw-compensated speech), as the jaw and tongue tip function as a synergy. This strategy, similar to patterns observed in individuals with Amyotrophic Lateral Sclerosis (ALS; Mefferd and Dietrich, 2020; Rong, 2019; Rong and Green, 2019), may support intelligibility at the word level but reduces clarity in connected speech due to the slower-moving jaw. Similarly, in younger children (e.g., two-year-olds), the lower lip often moves in sync with the jaw rather than independently. A child might produce /f/ only in jaw-assisted contexts, such as during transitions from an open-to-closed position (e.g., “off”) or closed-to-open (e.g., “fan”), but struggle with words like “fit” or “fish,” where the jaw remains fixed in a high-vowel position, which can result in substitution errors like “p” for “f” (e.g., /pɪʃ/ for “fish”).

In contrast to the typically developing child, a child could simply be lagging in speech motor development for their chronological age. For example, if a 5-year-old child is unable to elevate the tongue tip (which typically occurs by 3 years) we could potentially suggest a “delay in speech motor development” similar to how clinicians use the term “speech sound delay” for delays in acquisition of consonants/vowels, or refer to delays in physical motor development (e.g., crawling, sitting and walking). Indeed, speech motor delay is likely to result in speech sound delay. This perspective aligns with Kent’s concept of developmental functional modules (DFMs), which are semi-autonomous systems integrating structural, functional, and developmental elements to support vocalization and speech (Kent, 2022). While we adhere to the framework of synergies and gestures from articulatory phonology (Browman and Goldstein, 1992; Goldstein and Fowler, 2003; Hagedorn and Namasivayam, 2024; Namasivayam et al., 2020) rather than DFMs, both approaches highlight that different functional speech motor ensembles develop on distinct timelines, influencing speech production. Recognizing these movement patterns clinically is crucial.

## 3 Critique of a current classification model

### 3.1 The problem with ascribing observations in speech output and characterization of subgroups to cognitive-linguistic causes

It is widely acknowledged that children with SSD are not a homogenous group, and several subtypes of SSDs have been proposed. For some children with SSD, an “organic” or known etiology can be identified, such as hearing loss, cleft palate, or cerebral palsy, but for the majority of children with SSD there are no detectable causal factors or etiology (i.e., idiopathic). Interest in SSD subtyping has grown since the 1990s, with the two models now widely used being Shriberg’s Speech Disorders Classification System (SDCS; Shriberg et al., 2005, 2010; Shriberg and Wren, 2019) and Dodd’s MDD (Bradford and Dodd, 1996; Broomfield and Dodd, 2004; Dodd, 1995; Dodd, 2014). The SDCS follows an etiological/medical approach, categorizing speech disorders into Speech Delay, Motor Speech Disorders, and Residual Speech Errors, each with subtypes. The MDD classifies subtypes as Phonological Delay (PD), Consistent Phonological Disorder (CPD), Inconsistent Phonological Disorder (IPD), and Articulation (ARTIC), with Childhood Apraxia of Speech (CAS) later added as a distinct category (Bradford and Dodd, 1996; Cleland et al., 2025). Comprehensive reviews and critiques of these and other models can be found in Diepeveen et al. (2022), Maassen and Terband, (2024), and Waring and Knight (2013).

Studies suggest that subtypes of children within the MDD approach exhibit distinct profiles in input, output, cognitive, and executive functioning when completing tasks targeting these functions (Dodd, 2011, 2014). There is, of course, no doubt that input processing (e.g., hearing and speech perception issues; Hearnshaw et al., 2019; Rvachew, 1994) or output processing (e.g., neuromotor issues following traumatic brain injury, dysarthrias, cerebral palsy) may be causally related to speech symptoms. However, we argue that ascribing speech errors to the cognitive-linguistic domain (i.e., knowledge of higher-level phonological categories; Munson et al., 2005a, 2005b; Richtsmeier, 2010) is misguided. Dodd (2014) cites four studies in support of the claim that IPD, CPD, and PD all have different and specific cognitive-linguistic underlying issues (strength-weakness profiles; Dodd et al., 2010; Dodd and McIntosh, 2008, 2010; Dodd, 2011). This conceptualization has significant issues. According to Dodd and colleagues (Dodd et al., 2010; Dodd and McIntosh, 2008, 2010; Dodd, 2011), differences in speech errors originate from different subtle limitations in cognitive functions. However, in contrast to Dodd’s view that cognitive (in)flexibility directly causes these errors, we propose that components of cognitive flexibility (such as working memory) interact with limited speech motor skills to produce speech errors, as we explain below.

Dodd claims that children with CPD have underlying issues in rule abstraction and cognitive flexibility (Broomfield and Dodd, 2004, 2011; Dodd, 2005, 2014). Cognitive flexibility, as defined in recent work (e.g., Dajani and Uddin, 2015), entails several processes such as salience detection and attention, working memory, inhibition, and switching. Tests such as the Flexible Item Selection Task (FIST; Jacques and Zelazo, 2001) and the non-linguistic rule learning task reported in the studies by Dodd (2011, 2014) fail to delineate which of these processes are involved in these children. Studies indicate that children with phonological impairments often face working memory challenges (e.g., Farquharson et al., 2018; Waring et al., 2017) or difficulties with interactions between short-term and long-term memory (Couture and McCauley, 2000). Working memory is essential for managing sensory inputs, feedback monitoring, and motor output stability (Dromey and Benson, 2003; Levelt, 1983; Roberts et al., 2023). From a speech motor skills perspective, children with less developed speech motor skills may strain their working memory more due to additional resources necessary for input processing and monitoring to maintain motor stability and accuracy (Maxwell et al., 2003). Support for this claim comes from research that shows when working memory resources are taxed, such as during dual tasking or when performing unskilled movements requiring close attention, motor variability increases (Kleinow and Smith, 2000; Namasivayam and Van Lieshout, 2011).

Regardless of whether there exist working memory difficulties per se or a decrease in resources available for working memory (due to task complexity/dual tasking), the motor system responds in certain ways to reestablish stability in the system. This may be done by slowing down [increasing transition time to facilitate sensory feedback processing (Namasivayam and Van Lieshout, 2011; Terband and Maassen, 2010; Terband et al., 2014)], recruiting stabilizing movements (i.e., through intrusion gestures; Goldstein et al., 2007; Slis and Van Lieshout, 2016a,b), recruiting or freezing articulatory degrees of freedom (Buchanan and Kelso, 1999; Namasivayam et al., 2020), or by omitting or adding entire segments to facilitate stability. For example, in a CVC sequence, an increase in stability can be achieved by either omitting a final coda consonant, which will result in universally more stable in-phase (synchronously) coordinated CV segments alone or by adding a vowel, which would result in the same in-phase coordinated CV pattern across two syllables CV.CV, rather than involving the more challenging anti-phase (sequentially) coordinated movements required for a VC sequence (Goldstein et al., 2007; Namasivayam et al., 2020; Pouplier, 2008). We contend that this applies to children with consistent “phonological” impairments, where their errors likely stem from the interplay between constraints in working memory and limited speech motor skills. The resulting symptoms reflect the speech motor system’s struggle to achieve and maintain stability under these conditions.

For cognitive (in)flexibility to be considered the causal mechanism underlying CPD, it is essential that interventions specifically targeting this cognitive mechanism result in normalization of “phonological” error symptoms. The assertion that CPD stems from cognitive inflexibility (Broomfield and Dodd, 2004, 2011; Dodd, 1995, 2005, 2014) lacks empirical support. A hypothesis positing cognitive inflexibility as the underlying cause of speech errors, characterized by limitations in salience detection, attention, working memory, inhibition, and cognitive switching (Dajani and Uddin, 2015), would logically imply that improving these cognitive domains should yield significant decreases in phonological error patterns. However, to date, no such empirical mechanistic validation for these claims exists.

### 3.2 Limitations of cross-sectional studies in capturing developmental change

A corollary of the above point is that there is a risk of attributing causality to things that are simply correlated. Due to the cross-sectional nature of studies underlying the categorization of subtypes in the MDD approach, it is possible that cognitive-linguistic issues claimed to underlie speech symptomatology may simply be evidence of independent parallel development of speech-language and cognitive systems. There is strong evidence to suggest that cognitive flexibility and its components (working memory, inhibition/executive functioning, salience detection and attention, and switching) follow different and protracted developmental trajectories (Dajani and Uddin, 2015). Cognitive flexibility begins to develop in early childhood and increases around 7–9 years and matures around 10 years of age, while working memory emerges in early toddlerhood and improves into adolescence (e.g., Dajani and Uddin, 2015). Thus, if we sample cross-sectionally children who may be delayed in the independent trajectories of both cognitive and speech development, we may mistakenly assume a causal relationship between the two delays (see section 4.1 for more on these data sampling issues and its relation to categorical vs. dimensional explanations). To our knowledge, the data to fully support the above-mentioned perspective do not exist. In general, these considerations, when taken together, weaken the claim that the behaviors/symptomatology noted in children with (consistent) phonological disorder are truly or purely “phonological” in nature.

### 3.3 The fallacy of cognitive-linguistic intervention success as diagnostic validation

Because cognitive-linguistic intervention approaches (such as minimal pairs, maximal contrast, and other complexity approaches; Wren et al., 2018) are effective for idiopathic SSDs (McLeod and Baker, 2017), can we assume that the cause of the SSDs is higher-order cognitive-linguistic? This reasoning constitutes the fallacy known as “affirming the consequent.” The fact that speech errors can be resolved with cognitive-linguistic approaches to intervention (Wren et al., 2018) does not necessarily indicate that the origin of these errors is cognitive-linguistic. One might wonder, however, how these “phonetic/motor” errors, as argued in this paper, resolve with “phonological” intervention approaches. A partial answer to this can be found in the concept of stimulability. Stimulability (i.e., a child’s ability to detect and self-correct errors during imitation of the clinician’s speech model) may be one of the best predictors of outcomes and speech normalization in children with SSDs (McAllister et al., 2022; Miccio et al., 1999; To et al., 2022). In fact, most children are capable of normalizing speech errors by late school age, either through any type of treatment or simply through maturation (To et al., 2022). This suggests that a child’s ability to self-monitor and error detect and correct (i.e., compare and map sensory input to accurate speech motor plan and/or output) is as much or perhaps even more important than the type of intervention provided. Additionally, although some of the interventions are labeled “cognitive-linguistic” in nature, many of them also integrate production practice, which inevitably improves outcomes (e.g., Almost and Rosenbaum, 1998; Bowen, 2023; McLeod and Baker, 2017; Wren et al., 2018).

### 3.4 Do subgroup-specific interventions improve accuracy?

To be clear from the outset: we are not suggesting that children with CAS or childhood dysarthria do not require distinct interventions—on the contrary, they clearly do (e.g., see Pennington et al., 2013; Preston et al., 2019). Ample evidence demonstrates that children with speech disorders benefit from specialized motor speech interventions (e.g., see Chapter 14 in McLeod and Baker, 2017). Our concern lies with the conceptualization and use of the argument that “intervention targeting subgroups’ specific weaknesses [will] lead to improved accuracy relative to other subgroups of SSD” (Table 1, Dodd, 2014, p. 194) as evidence for the existence of phonological delay/disorder subgroups. Intervention should undoubtedly be aimed at strengths/weakness profiles; however, the authors define these strengths and weaknesses in the phonological domain and use this as a critical pillar to support MDD subtyping. They use four key references in support of this: Broomfield and Dodd (2011), Crosbie et al. (2005), Dodd et al. (2008), and Dodd et al. (2010). These references, in our view, offer only limited or anecdotal evidence and may involve misinterpretations of the data. Below, we briefly examine these four pieces of evidence cited in Dodd (2014).

The strongest evidence that Dodd (2014) claims for a differential effect of intervention on SSD subtype is a randomized controlled trial (RCT) study by Broomfield and Dodd (2011). Despite the RCT design, a deeper look reveals serious methodological issues, including the use of a non-standardized in-house oromotor skill rating scale with no published psychometric data to differentiate diagnostic categories. It is unclear whether the three diagnostic categories (comprehension, expression and speech deficits) tested in this study in fact map onto MDD subtypes. Moreover, the “speech” category used included both “articulation” and/or “phonology” (Broomfield and Dodd, 2011, p. 632), creating a potential subtype confound. The study’s notable strength was its large sample size (*n* = 730), and the data showed a significantly better outcome in the intervention group (children receiving phonological contrast and core vocabulary therapy) compared to the no-intervention control. However, because the data were reported as z-score changes, the clinical and practical significance of these findings is not immediately clear [e.g., Are changes meaningful? Are changes outside of measurement error? Do changes reflect normalization? (Bothe and Richardson, 2011)].

Another concerning aspect is illustrated in Figure 3 (in Broomfield and Dodd, 2011, p. 634), which shows that while a statistically significant proportion of children who received interventions exhibited improved outcomes compared to those with no intervention, approximately 25% of the intervention group showed no change or worsened. While many clinical trial studies show non-responders to an intervention, these non-responders are evidence contrary to the author’s conclusion that the study offers “evidence supporting the case for the differential diagnosis model” (Dodd, 2014, p. 193). Nevertheless, this success rate of SSD intervention aligns with those observed in other studies (Maas et al., 2012; Namasivayam et al., 2021). Recent research suggests that children who do not benefit from traditional interventions might respond better to ultrasound biofeedback approaches (McAllister et al., 2022), supporting a more objective instrumental-based precision medicine-like approach for children with SSD, who are traditionally considered “therapy-resistant.” Also, we remind the reader that speech errors being resolved with specific phonological interventions does not necessarily indicate that the origin of these errors is phonological, and that factors such as stimulability, speech production and sensory-motor practice likely play a substantial role (see “affirming the consequent” problem, above).

The next piece of evidence put forth by Dodd (2014) is the study by Crosbie et al. (2005). This study was conducted on 18 children with severe SSD, comparing phonological contrast intervention to core vocabulary therapy. This study utilized a multiple baseline design with alternating treatments. In the study, after a baseline period, children were assigned to one of the two interventions. The first intervention was followed by the other intervention with a 4-week washout interval between the two interventions. Each intervention period was 8–9 weeks long. They reported that core vocabulary therapy, when used with children exhibiting IPD, led to greater improvements in percent consonants correct (PCC) and inconsistency scores. In contrast, phonological contrast therapy, when used with children displaying CPD, resulted in better outcomes for the same variables. However, despite the purported validity of these data, a significant issue with the data analyses is evident in Table 2 (in Crosbie et al., 2005; p. 483). The raw data in Table 2 reveal that the claimed benefits of core vocabulary therapy for the IPD group are driven by just two of the seven participants (Participants 1 and 2). Interestingly, without these two participants, the change in the PCC scores for the IPD group receiving core vocabulary intervention first (mean = 9.4 and SD = 4.6) is lower than the group receiving phonological contrast intervention first (mean = 10.7; SD = 5.5), contrary to what is claimed in the paper. Furthermore, the observed improvement in inconsistency scores for IPD children who received core vocabulary intervention versus phonological contrast therapy is likely an artifact of floor/ceiling effects and a regression to the mean. Since the children with CPD were already at “floor” scores initially (as noted in Table 2, p. 483), they had limited potential for further reduction in inconsistency with intervention (e.g., 24% inconsistent).

The third study cited by Dodd (2014) as evidence for differential effects of intervention on SSD subtypes is the research conducted by Dodd et al. (2008). This study compared minimal versus maximal contrast approaches for children with phonological disorders, rather than SSD subtypes as discussed in the MDD approach. While children showed progress in therapy with improvements in speech accuracy and a reduction in error patterns, there was no significant difference in the progress made between those receiving minimal or maximal contrast interventions. This finding does not support the notion that subtypes of SSD differ on outcomes based on type of intervention, nor does it suggest that response to intervention is different when type of phonological intervention is altered.

Lastly, Dodd (2014) lists as evidence a non-peer-reviewed textbook chapter (Dodd et al., 2010, p. 124). This textbook chapter consists of a summary of statistically weak non-experimental study designs (e.g., case studies), post-hoc retrospective analysis (Forrest et al., 1997) and one randomized controlled trial (RCT; Broomfield and Dodd, 2011, discussed above), and overall constitutes low evidence for differential effects of intervention on SSD subtypes. To summarize, the four studies cited by Dodd (2014) have notable weaknesses that limit their support for differential intervention effects on SSD subtypes.

### 3.5 Popularity and sales as a misleading indicator of validity

Included in Dodd’s (2014) summary of evidence for MDD approach to classification (Table 1, p. 194) is the feasibility of the MDD system based on increased clinical/research use and higher international sales. Dodd (2014), in the evidence summary Table 1, states that “Sales figures [of the DEAP test; Dodd et al., 2002] indicate increased use internationally since publication.” Ttofari Eecen et al. (2019, p. 689) takes this one step further, claiming, “The high uptake of this assessment tool by speech-language pathologists provides some evidence for the face validity of the classification system.” The claim that the high quality of the assessment tool is attested by its high uptake and that its high uptake is due to that high quality relies on circular reasoning and constitutes a false cause fallacy. This logic is flawed, as high-uptake of an assessment tool is driven by a multitude of factors at the level of the clinician (familiarity of test, analog vs. digital version availability, clinician preference, norming data availability), resources (e.g., budget restrictions, publisher accessibility), and policy (national policies based on consensus and feasibility; Cleland et al., 2025; Ttofari Eecen et al., 2019). Perhaps most importantly, high uptake and popular use are in no way indicators of scientific validity. In fact, the concept of face validity is notably absent from the 2014 standards and guidelines documentation on validity produced by three leading national organizations: the American Educational Research Association, the American Psychological Association, and the National Council on Measurement in Education (Royal, 2016). Consequently, the argument based on face validity is among the weakest scientific justifications for the MDD approach.

### 3.6 Classification models lack comprehensive speech motor testing

The original Dodd’s MDD is a descriptive-linguistic classification system that is based on data from clinical observations (Dodd et al., 1989) and theoretical modeling (Ttofari Eecen et al., 2019). Much of these data are filtered through the DEAP test (Dodd et al., 2002; Dodd, 2005), which uses an oral motor screening (DEAP; p. 39) and a case history procedure to identify the presence of underlying speech motor disorders. Additionally, subgroup classification of “Oral Motor Disorder” (DEAP; p. 116) was based on procedures that considered facial symmetry, presence of drooling, muscle tone, breath control, facial palsy, progress in speech therapy, difficulty sequencing oral-motor movements, etc. While we acknowledge that these procedures are likely to detect disorders with overt neurological involvement (e.g., dysarthrias), as discussed in Section 2.2.2, the DEAP tasks—such as diadochokinetic (DDK) testing, phoneme sequencing, non-speech movements, verbal fluency, and intelligibility—lack the sensitivity required to identify more nuanced motor-based speech limitations. These include jaw sliding, adaptive jaw compensation, undifferentiated tongue gestures, and covert motoric contrasts (Cleland et al., 2017; Gibbon, 1999; Mogren et al., 2022; Namasivayam et al., 2020; Terband et al., 2013; Ziegler, 2016). As a result, children who pass the DEAP oromotor screening may be misclassified as having SSDs of cognitive-linguistic origin, such as phonological delay or phonological disorder, despite presenting with subtle speech motor skill limitations.

Two recent studies have attempted to validate the MDD. Dodd et al. (2024) faced the same issue of relying solely on oral-motor screening within the DEAP test to identify and categorize participants. This constitutes circular reasoning, once again, involving using a test designed to identify certain subtypes of SSDs to validate the existence of such subtypes. To avoid this type of circular reasoning, Ttofari Eecen et al. (2019) also aimed to validate Dodd’s MDD classification system but based their evaluation on standardized speech motor testing that is not designed to fit the MDD categories [Verbal Motor Production Assessment in Children (VMPAC); Hayden and Square, 1999]. While VMPAC captures aspects like jaw sliding, jaw excursion range, and jaw-assisted movements, it still fails to detect other subtle issues, such as undifferentiated tongue movements and covert speech motor contrasts (Gibbon, 1999; Mogren et al., 2022; Namasivayam et al., 2020). Therefore, the current evidence in support of MDD lacks adequate sensitivity to subtle speech motor issues, unlike instrumentation-based methodologies (Sugden and Cleland, 2022). We recommend future subtype validation attempts also include instrumentation-based methodologies (e.g., EPG, ultrasound data) as outlined in the recommendation section (Sugden and Cleland, 2022). Unfortunately, other models that attempt to classify children with SSD, such as the Speech Disorders Classification System (SDCS; Shriberg et al., 2010) and psycholinguistic frameworks, such as the Stackhouse and Wells’s (1997) framework and Process-Oriented Profiling of Speech Sound Disorders (Diepeveen et al., 2022) are plagued by the same limitations concerning their dependency on subjective perception and transcription-based methods as noted above (Waring and Knight, 2013). Since none of these models have reached the same clinical impact as the MDD classification system (as mentioned in section 3.5), we will continue to focus on the latter approach.

### 3.7 Cross-language comparisons: do they validate subgroups?

Perceptual data used in support for one of the theoretical pillars for the MDD classification system is that similar subgroups (IPD, PD, CPD) have been identified across multiple languages in similar proportions (Dodd, 2014; Hua and Dodd, 2000; Ttofari Eecen et al., 2019). However, data from cross-language comparisons fail to validate the construct of subgroups, as they suffer from the same pitfalls as studies based on English-speaking children, discussed previously (see sections 3.1 to 3.6). These pitfalls, as mentioned earlier, range from flawed cross-sectional studies, misattribution of intervention success to phonological causes, and using popularity as a misleading indicator of validity, to inadequate speech motor testing leading to misclassification.

Interestingly, Hua and Dodd (2000) claim that the presence of cross-linguistic differences in acquisition and error patterns cannot “be explained by appealing to the biological constraints or articulatory limitations of young children” (p. 37). Dodd argues that the rarity of alveolo-palatal affricates in Putonghua (Modern Standard Chinese) and the earlier acquisition of affricates compared to English-speaking children cannot be explained by the frequency of these phonemes in the language or by articulatory/biological constraints. Instead, Dodd suggests that these differences are linked to the functional load and phonological saliency of the segments (Dodd, 2014; Hua and Dodd, 2000). We do agree that saliency and functional load of a phoneme in a child’s ambient language are important; however, recent acoustic and transcription data from Ma et al. (2022) show that even within acquisition of affricates, alveo-palatal affricates in the Putonghua language that use the tongue body are acquired earlier than alveolar and retroflex affricates. This supports the oromotor maturation hypothesis, as it is relatively easier for children to control the muscles related to the elevation of the tongue body (as it matures earlier) than those used to raise the tongue tip (Kent, 2021; Ma et al., 2022). Further support for this argument comes from Li and Munson (2016), who present evidence that the acquisition of fricatives in Putonghua aligns with articulatory constraints—offering empirical backing for the claims advanced in this section. They also highlight that Hua and Dodd’s conclusions were based on a limited set of word types, which may have introduced interpretive bias. This suggests both oromotor maturation and ambient language play a role in the acquisition of phonemes, and that previous studies (e.g., Hua and Dodd, 2000) that have derived acquisition data based on only IPA transcription and no instrumental data may have missed subtle contrasts due to listeners’ categorical perception biases (Kent, 1996; Li, 2012; Li and Munson, 2016; Li et al., 2009; Ma et al., 2022; Mowrey and MacKay, 1990). These insights suggest that the speech sound acquisition process is more complex than previously thought and involves a dynamic interplay of cognitive development, ambient language, and oromotor maturation (Green and Nip, 2010; Kent, 2021; Ma et al., 2022; Namasivayam et al., 2020; Nip et al., 2011; Noiray et al., 2019a,b). Therefore, the current evidence does not robustly support the existence of distinct subgroups in SSD based on phonological error patterns but rather points to a multifaceted understanding of speech sound acquisition. This underscores a more nuanced understanding of speech sound acquisition, suggesting that the speech errors used to differentiate subtypes of MDD are better interpreted as reflections of a gradual and dynamic cognitive and oromotor development in the context of the characteristics of the ambient language, rather than evidence of discrete subgroups (Maassen and Terband, 2024; Namasivayam et al., 2020).

## 4 The case for a dimensional approach to SSD classification

### 4.1 The butterfly problem: when subtypes are sampling artifacts

Broomfield and Dodd (2004) provided prevalence rates for the subtypes (PD, CPD, IPD, and ARTIC) based on data from 320 children with SSD as follows: 57.5% demonstrated PD, 20.6% “consistently made non-developmental errors” (i.e., CPD; p. 135), 9.4% “made inconsistent errors on the same lexical item” (i.e., IPD; p.135), and 12.5% had articulation disorder. These are similar to those reported earlier by So and Dodd (1994), where 47% of children demonstrated PD, 30% demonstrated CPD, 12% demonstrated IPD, and 11% had articulation disorder. No children were diagnosed with CAS in these studies. Although these disorder subtypes are currently clinically popular (e.g., see Cleland et al., 2025; Terband et al., 2019; Waring and Knight, 2013), there is one major fundamental flaw in how these classifications and subtypes were created. The assignment of these subtypes by Dodd and colleagues (Dodd, 2014; Hua and Dodd, 2000; Ttofari Eecen et al., 2019) or the Speech Disorders Classification System by Shriberg et al. (2010) is hindered by artifacts of cross-sectional sampling of a population. These subtypes arise from limitations of cross-sectional data and lack of longitudinal validation, as elaborated below.

Dodd and colleagues derived prevalence estimates using observational, descriptive cross-sectional study designs that assessed children at a single point in time. Such measurements are less than ideal for capturing the behavior, which can potentially change over time (Capili, 2021; also see section 3.2). Indeed, Dodd and colleagues (Dodd et al., 2003, p. 622) point this out when discussing the literature on normative data of children’s phoneme repertoire: “cross-sectional studies cannot trace the sequential development of phonemes” or developmental patterns in children.

Ironically, Dodd and colleagues, themselves, provide direct evidence that error patterns produced by children with SSD evolve over time due to intervention, developmental, and/or maturational effects (Dodd et al., 2018). Their 2018 study demonstrated that children diagnosed at 4 years of age with inconsistent atypical patterns (i.e., phonological disorder) were reclassified as exhibiting phonological delay/articulation disorder by age 7, while the speech difficulties of those initially diagnosed with phonological delay and articulation disorder generally resolved by age 7. Although the speech difficulties of fewer children with atypical speech error patterns resolved by age 7 (Dodd et al., 2018; Morgan et al., 2017), the change errors and eventual resolution suggest a developmental course of speech error resolution (see Table 4 in Dodd et al., 2018, p. 95). As speech-motor skills develop, severity typically declines and errors resolve. The subtype categories noted by Dodd et al. are a sampling artifact of cross-sectional designs. Strong evidence from multiple large studies with extensive datasets, including cross linguistic research, shows that most children with idiopathic SSD resolve their speech errors by ages 7 to 8, often earlier, with or without treatment (Morgan et al., 2017; Shriberg, 1994; To et al., 2022; Wren et al., 2016). This challenges the concept of distinct and categorical subtypes of idiopathic SSDs as outlined in the descriptive-linguistic approach (Dodd, 2005).

**Figure 1:**
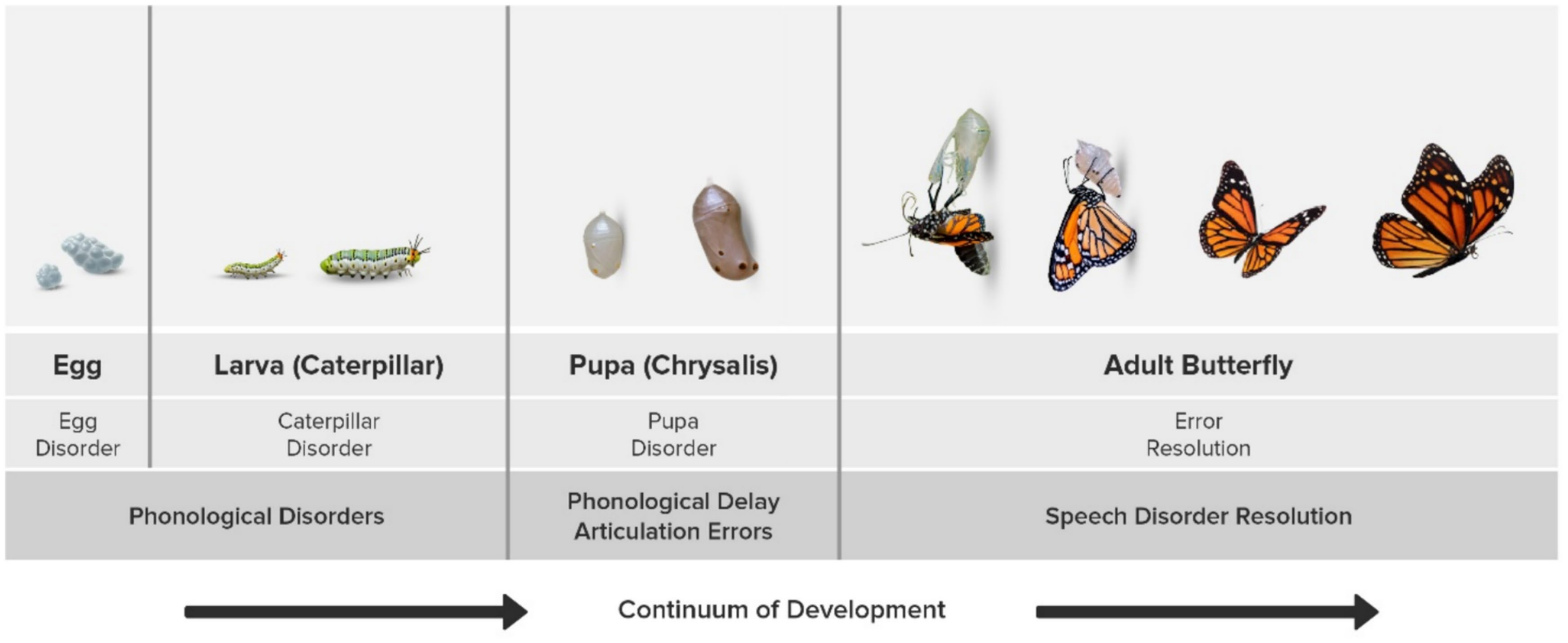
Rethinking SSD classification. Like the butterfly life cycle, cross-sectional sampling may mislabel developmental processes as distinct subtypes, while a longitudinal perspective recognizes them as part of a continuous process.

We would like to illustrate this further with an example of the life cycle of a butterfly. If one were to cross-sectionally sample a population of butterflies over the lifespan, we would label each stage (egg, larva, caterpillar, pupa, adult butterfly) a separate species or subtype. However, if we examine the butterfly population longitudinally, a continuum of development would be evidenced (Figure 1), much like the continuum of speech error pattern resolution that we propose (as a function of improving speech motor skills). Simply stated, in a butterfly life cycle the caterpillar is not a disorder!

As professionals in this field, we are called to consider a shift in our clinical perspective—from viewing disorders as categorical entities to understanding them along a continuum of severity. This dimensional approach aligns with contemporary frameworks in rehabilitation science and the American Psychiatric Association’s Diagnostic and Statistical Manual of Mental Disorders (DSM-5; American Psychiatric Association, 2013), as discussed below.

### 4.2 Categorical vs. dimensional

Apart from the large volume of data that suggests the natural developmental progression of error types, the call for a shift in perspective from categorical to dimensional is also fueled by the failure of the categorical approach at multiple levels. There have been multiple attempts by different groups of researchers to identify distinct psycholinguistic speech-processing deficits or create diagnostic markers for different speech disorder subtypes (e.g., Diepeveen et al., 2022; Shriberg et al., 2017). Despite the large volume of complex work in this area to date, there have been no conclusive single marker or a set of markers specific and/or sensitive enough to identify each disorder subtype (e.g., Shriberg et al., 2017; Shriberg and Wren, 2019). Problems with using discrete speech disorder subtypes are also evident from the overlap of speech error types between different SSD labels, interpretations based on subjective clinical experience rather than objective data (Diepeveen et al., 2022), the complexity of test battery assessments (Shriberg et al., 2010), the lack of required sensitivity and specificity levels even in published tests (e.g., Strand et al., 2013), errors that are subclinical and generally not observable without instrumentation (Diepeveen et al., 2022; Gibbon, 1999; Kent, 1996; Namasivayam et al., 2020; Richtsmeier, 2010; Vick et al., 2014), lack of definitions and standardized objective criteria (Iuzzini-Seigel et al., 2017), poor reliability of categorical diagnosis of SSDs even with expert clinicians (Murray et al., 2024), and challenges in effectively communicating labels to families (e.g., Bishop et al., 2016; Csercsics et al., 2024) have collectively resulted in a reluctance to adopt the labels of SSD subtypes clinically (e.g., see Csercsics et al., 2024). Despite these well-known issues, there has been a call for the widespread use of subtype labels from the MDD (Dodd, 2014) within the UK’s National Health Service, (Cleland et al., 2025). There is a serious issue of circular reasoning here. If the educational environment for SLPs—through textbooks, curricula, and clinical training—already endorses and teaches the use of subtype labels from the MDD (Dodd, 2014), then surveying SLPs who have been exclusively trained within this framework creates a biased sample. Their preference for the same model (MDD) is not an independent validation but rather a predictable outcome of their training. Using such responses to justify and solidify service delivery practices based on this model reinforces existing assumptions rather than objectively evaluating alternative approaches. Developing unbiased methods to determine service delivery pathways is necessary, though beyond the scope of this paper.

A dimensional perspective may help address the aforementioned challenges. For example, the DSM-5 (American Psychiatric Association, 2013) includes both categorical and dimensional models for diagnosis of mental health conditions. The latter is mentioned in its chapter called Emerging Measures and Models. Some psychiatric conditions are conducive to categorical labels (e.g., Schizophrenia), while others, such as personality disorders, have been approached from a dimensional perspective. We do agree that for some speech issues following neurological damage or structural impairment like Childhood Dysarthria or cleft lip/palate, a categorical label is most suitable, particularly in cases of well-defined causes and symptoms. However, for idiopathic SSDs such as CPD, IPD, and PD, where a continuum of severity and symptoms is observed (refer to Table 4 in Dodd et al., 2018, p. 95), many of the labeling and diagnostic issues mentioned earlier could be addressed by adopting a dimensional labeling system. Importantly, within this dimensional approach, speech error patterns (e.g., fronting, backing, stopping, etc.) would represent the lower extreme along a continuum of typical speech development. We emphasize the term “typical” because this continuum reflects the natural developmental progression of error patterns toward potential self-resolution, as noted by several researchers (Dodd et al., 2018; Morgan et al., 2017; Shriberg, 1994; To et al., 2022; Wren et al., 2016). The dimensional approach is compatible with a trend identified by Duchan (2005), in which clinicians are moving away from classifications based on etiology and toward assessments that identify areas of competence and incompetence (which may be viewed in terms of strengths and weaknesses). Children with idiopathic SSDs may likely occupy the low end of the speech motor skill continuum, similar to arguments made for stuttering (Namasivayam and Van Lieshout, 2011; Namasivayam et al., 2020; Van Lieshout et al., 2004). The next section elaborates on this notion.

### 4.3 Is it a difference or a disorder? Speech errors as adaptive and compensatory strategies

Namasivayam et al. (2020) introduced the dimensional speech motor skill approach to SSD. This approach examines the potential speech motor underpinnings of SSDs through the lens of the articulatory-phonology (AP) framework (Browman and Goldstein, 1992; Gafos and Goldstein, 2012). Based on a comprehensive review of behavioral and articulatory kinematic data, the authors propose an alternative dimensional perspective, proposing that speech sound errors in children with SSDs arise from a complex interplay among articulatory gesture dynamics, an immature speech motor system with constrained motor capacities, and boundary conditions imposed by physical, physiological, cognitive and functional constraints vis-à-vis the language(s) being learned. The view posits that the different types of speech sound errors observed and their changes over the development trajectory may simply reflect variations in how individuals develop strategies to manage the challenges of limited speech motor skills.

According to this view, many of these speech errors may be viewed as compensatory adaptations aimed at increasing speech motor stability. These adaptations include increased movement amplitude, reduced speech rate, tongue bracing, intrusion gestures, deletions at the segmental, gestural, or syllabic levels, and increased phase lag between movements of articulators. These strategies are observed in both typical and disordered speech production to improve speech motor stability and intelligibility when one is at the low end of the speech motor skill continuum (Fletcher, 1992; Namasivayam and Van Lieshout, 2011; Namasivayam et al., 2020; Van Lieshout et al., 2004). This view is a drastic departure from traditional characterizations of the presence of speech sound errors as speech sound “disorders.” In this alternative view, speech sound errors in idiopathic SSDs can be considered as differences in speech motor strategy and compensatory adaptations rather than a disorder or deficit.

To illustrate the gesture and synergy perspective proposed by these authors (Namasivayam and Van Lieshout, 2011; Namasivayam et al., 2020; Van Lieshout et al., 2004), consider the well-established concept that the upper lip, lower lip, and jaw act as a functional synergy during target bilabial segments (e.g., production of /p/), while the jaw and tongue form a similar synergy during target alveolar constrictions (e.g., production of /t/) (Kelso and Tuller, 1983). Within these functional synergies, if one articulator is restricted or impaired, other articulators compensate to achieve the intended task-level goal. These compensatory adjustments occur automatically, on a reflex-like timescale, through dynamic self-organization, as demonstrated in studies involving unexpected articulatory perturbations (Kelso, 1995; Kelso and Tuller, 1983). For example, if lower lip movement is restricted during bilabial closure, the upper lip and jaw increase their movement range to compensate. Similarly, if jaw movement is constrained such as by a bite block or an object held between the teeth, the upper and lower lips adjust their amplitude to achieve bilabial closure. Likewise, when fine tongue control is limited, the jaw often compensates by supporting tongue elevation (e.g., Rong and Green, 2019).

Evidence from adult neurodegenerative research reinforces this compensatory principle. In early-stage bulbar ALS, reduced tongue control leads to increased jaw movement as an adaptive strategy to preserve intelligibility. However, this compensation breaks down in later stages, resulting in a sharper decline in speech intelligibility (Mefferd and Dietrich, 2020; Rong, 2019; Rong and Green, 2019). Importantly, compensation may not be limited to the tongue-jaw pair but may involve broader articulatory synergies, as shown in studies using kinematic consonant distinctiveness space measures (Teplansky et al., 2023). This phenomenon is likely driven by neuroplasticity, supported by findings of increased functional connectivity in early symptomatic ALS compared to typical controls using functional magnetic resonance imaging and magnetoencephalography (Dash et al., 2024; Konrad et al., 2002; Lulé et al., 2007, 2009; Proudfoot et al., 2018).

Applying this framework to children with SSDs, it is well-documented that many of these children exhibit undifferentiated tongue gestures (Gibbon, 1999) relative to typically developing children (Kabakoff et al., 2021). Given this, it is unsurprising that a high prevalence of jaw sliding (anterior or lateral) is observed in this population, likely representing an adaptive response to reduced tongue control (Mogren et al., 2020, 2022; Namasivayam et al., 2013, 2020; Terband et al., 2013). Conversely, in cases where jaw stability is compromised, some children develop compensatory strategies such as clenching their teeth together and retracting their lips, engaging facial musculature to stabilize the jaw and facilitate greater freedom of movement for the lips and tongue (Mogren et al., 2020, 2022; Ward et al., 2013). Currently, the field lacks a fully developed dimensional model for children with idiopathic SSDs that offers systematic and stepwise definitions for navigating the continuum of speech motor skills. We hope that this paper highlights the potential of such a model as an alternative to existing categorical approaches, suggesting it could spur the development of new scales and methodologies to assess speech motor skill across the entire range of its development (Namasivayam et al., 2020).

## 5 Where do we go from here?

The premature standardization of categorical labels in healthcare and policy based on models such as the MDD (Dodd, 2014; Hua and Dodd, 2000) and the Speech Disorders Classification System (SDCS; Shriberg et al., 2010) may not adequately serve the nuanced needs of the children in our care. With advances in instrumental phonetics and a growing emphasis on precision medicine, a more evidence-based diagnostic framework for SSD is increasingly attainable. It is imperative that we pursue approaches that reconcile current clinical practices with instrumental insights into speech motor development. The following section outlines potential strategies to support this shift.

## 6 Recommendations

### 6.1 Advocating for a dimensional approach

**Figure 2:**
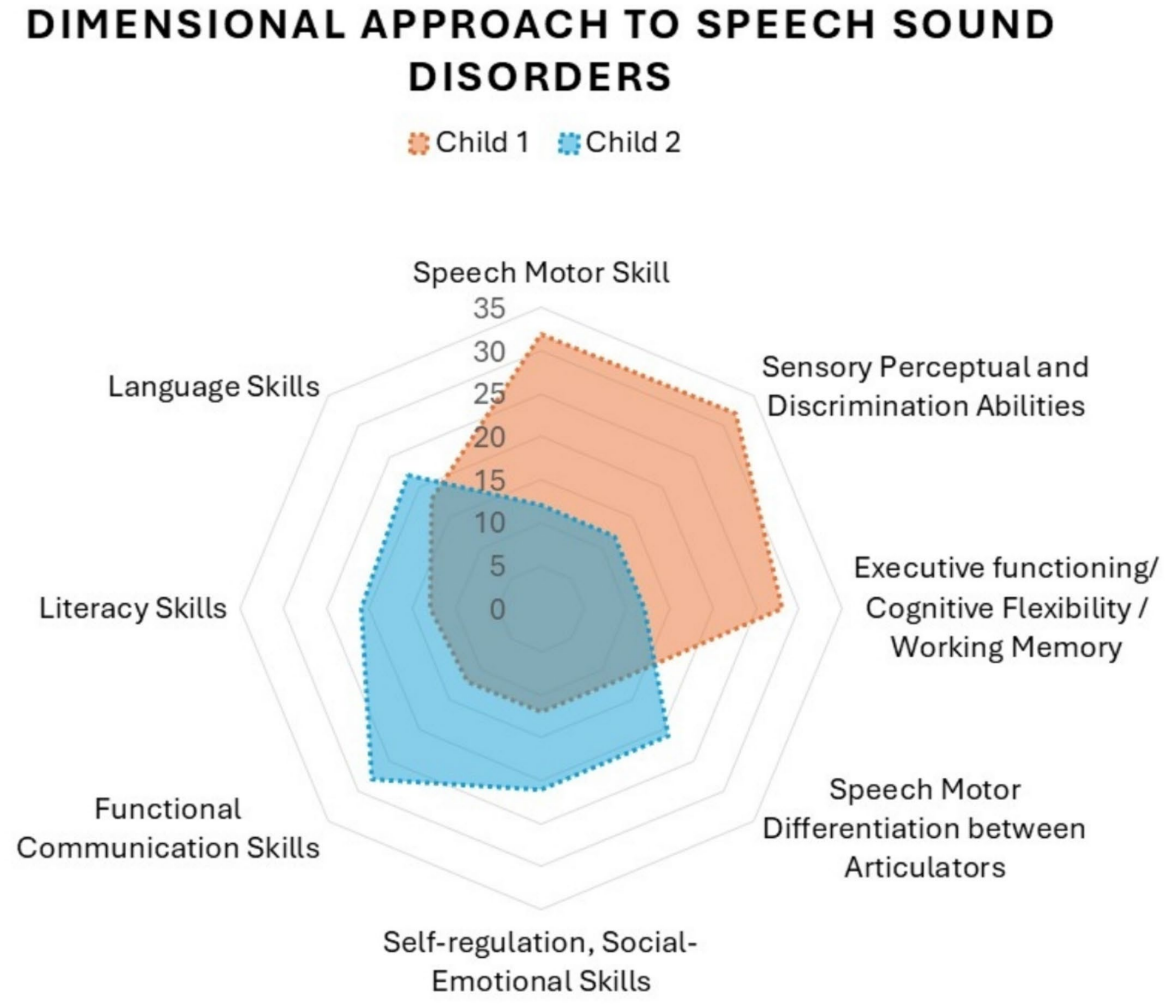
A dimensional framework for SSD assessment. Evaluating strengths and areas for improvement across cognitive, linguistic, sensory-perceptual, sensory-regulation, social–emotional, literacy, speech motor and functional communication domains to capture the full spectrum of development in-line with the levels proposed by World Health Organization’s (WHO’s) International Classification of Functioning, Disability and Health–Children and Youth (ICF-CY) version (World Health Organization, 2007).

What is an alternative to using labels based on descriptive-linguistic approaches for idiopathic SSDs? We advocate for moving beyond the traditional approach of conducting a basic oro-motor screening followed by a one-dimensional, IPA-based auditory-perceptual evaluation (e.g., DEAP test; Dodd et al., 2002) and selecting intervention targets based on age norms (McLeod and Crowe, 2018). Instead, we advocate for a comprehensive assessment framework that examines a child’s strengths and weaknesses across multiple domains, including cognitive flexibility (e.g., working memory), perceptual and discrimination abilities, speech encoding and sequencing skills, vocabulary (inventory of memory-stored auditory-motor word forms) and the degree of speech motor differentiation among articulators (Figure 2; see Appendix A for articulator differentiation timelines and potential assessments in Supplementary material). Such a dimensional approach has been proposed recently for Autism (Frazier et al., 2023; Moore et al., 2024). The dimensional approach eliminates the need for rigid diagnostic labels such as “phonological disorder” (Maassen and Terband, 2024) and instead allows clinicians to describe specific strengths and challenges to guide intervention in areas requiring support. See Appendix A for a hypothetical case (with different levels of description) illustrating how these profiles can be used clinically. A structured model reflecting this perspective has been recently proposed by Maassen and Terband (2024).

Within this framework, we also recommend moving away from age-based norms, which can be misleading (Lof, 2004). Normative charts on speech sound acquisition illustrate the ages at which children achieve different levels of mastery (e.g., 50, 70%, or 90% accuracy). However, this can lead to misinterpretation. For instance, McLeod and Crowe (2018) report that the lower boundary (50% criterion) for /s/ is approximately 36 months, which might be mistakenly interpreted as the age at which /s/ first emerges. In reality, this threshold indicates that by 36 months, 50% of typically developing children have already mastered /s/ (Lof, 2004). Delaying intervention until a child reaches a given age threshold places them at a disadvantage from the outset (Lof, 2004). Thus, rather than relying on age norms to select speech intervention targets (McLeod and Crowe, 2018), we propose an approach grounded in a child’s ability, stability, and stimulability of the underlying sounds and speech motor system. For instance, instead of introducing /s/ based solely on normative age charts, clinicians should assess whether the child understands the properties of /s/ (e.g., that it is a fricative and contrasts with other sounds to convey meaning), whether the child can produce earlier-emerging fricatives (e.g., /f/), and what stage of speech motor development the child is in (see Table 1, Section 2.2.3). For example, if the child does not yet have independent control of the upper and lower lips for producing /f/, that skill should be targeted before moving on to mastering independent tongue tip elevation, and subsequently to more refined articulatory features such as tongue grooving required for /s/ (Hayden et al., 2020). This paper, along with the perspectives of Maassen and Terband (2024), supports a dimensional approach to SSD that aligns with contemporary rehabilitation science.

### 6.2 Use of speech science instrumentation to aid diagnosis

Cutler’s Native Listening theory argues that perception is tuned by native-language experience to optimize everyday communication rather than fine-grained phonetic analysis (Cutler, 2012). Consequently, sole reliance on categorical judgments by SLPs can obscure sub-phonemic patterns better revealed by instrumental approaches (e.g., ultrasound, electropalatography, acoustic and kinematic analyses), positioning them as complementary to, rather than replacements for, expert listening.

It is important to recognize that the accuracy of differential diagnosis using perceptual procedures, even among experts, remains low (e.g., Murray et al., 2024). To enhance clinical evaluation, diagnosis, and intervention, researchers have recommended the use of instrumental tools such as acoustic analysis (e.g., spectrograms), electropalatography (EPG), and ultrasound (e.g., Shriberg and Lof, 1991; Sugden et al., 2019). For instance, ultrasound-aided transcription has been shown to identify more instances of increased variability and abnormal timing errors as compared to audio-only transcription (Sugden and Cleland, 2022). Emerging technologies, such as markerless facial tracking, have also become increasingly popular for identifying and monitoring atypical facial movements in individuals with speech disorders (Bandini et al., 2017; Mogren et al., 2022; Palmer et al., 2024). While other fields have widely embraced technology and precision medicine, pediatric SSD diagnosis continues to rely heavily on subjective, behavioral, and perceptual assessments. However, objective instrumental approaches are now more accessible to clinicians than ever before. For instance, the cost of portable ultrasound devices has decreased to a level that is now accessible for many small to mid-sized clinical centers. Similarly, acoustic analysis of speech signals no longer depends on costly equipment and proprietary software, thanks to the availability of advanced, free tools such as PRAAT (Boersma and Weenink, 2025), as well as algorithmic approaches (e.g., data-driven, unsupervised subtype classification, Vick et al., 2014).

Instrumental assessment is gaining ground in several areas of practice in speech-language pathology. Protocols or guidelines for instrumental measures have been proposed for several disorders: voice (Patel et al., 2018), velopharyngeal function and resonance (Bettens et al., 2014; Perry and Schenck, 2013), and pediatric swallowing and feeding (Arvedson and Lefton-Greif, 2017). It is highly likely that artificial intelligence will play an increasing role in maximizing the practicality and value of instrumental procedures. Critically important in the development of instrumental assessments are: (a) large data sample across different age and gender groups, (b) flexible adjustment of analysis parameters to account for age and gender, and (c) specification of protocol, including measurement methods for various speech tasks. Combining auditory-perceptual and instrumental methods yields a multi-granular analysis in which different levels of observation can be compared and integrated. Automatic speech recognition (ASR) holds great promise for assessing speech sound disorders in children and for supplementing treatment (Benway and Preston, 2024), but a concerted effort should be made to compile publicly available datasets for children’s speech. A suitable launching point is a review of 88 studies representing more than 500 children, infants, toddlers, and adolescents (ages 0–17 years) with speech disorders in more than 10 countries (Usha and Alex, 2023). The authors lamented the limited availability of speech databases needed to develop ASR technology for children, noting that “the available literature is contained in databases that require either subscription or specific institutional credentials to have access” (p. 35053). To be sure, there is much work to be done to incorporate ASR in routine clinical practice for SSD, but the remarkable strides in ASR for adult speakers are encouraging.

### 6.3 Need for additional educational system reforms and clinical training

Clinical training of future speech-language pathologists should incorporate speech motor development (e.g., speech motor differentiation), cognitive factors (e.g., working memory), perceptual functions, vocabulary (stored word forms), and speech planning, and sequencing abilities into the assessment and treatment of speech sound errors. Additionally, clinicians must receive specialized training in cognitive-perceptual-motor architecture of speech production and instrumental phonetics, including the use of acoustic and ultrasound technology, as part of standard university curricula for speech-language pathology. To facilitate this integration, textbooks should include contemporary concepts on these topics, ensuring they become part of the core curriculum rather than being limited to continuing education or advanced professional development courses only available to a select few. For additional information and recommendations for how the field of Speech-Language Pathology can overcome the barriers of technological education (see Appendix B in Supplementary material).

### 6.4 What about the use of cognitive-linguistic intervention approaches?

We do not advocate for discontinuing cognitive-linguistic intervention approaches (such as minimal pairs, maximal contrast, and other complexity approaches; Wren et al., 2018) for children with SSDs altogether, given their long-standing clinical use, ease of implementation, and demonstrated effectiveness for some populations (McLeod and Baker, 2017). Nor do we suggest that approaches such as minimal pairs, maximal oppositions, or Hodson’s Cycles lack efficacy—numerous randomized controlled trials support their benefits (e.g., Almost and Rosenbaum, 1998; Wren et al., 2018). However, their effectiveness is likely not solely attributable to improved knowledge of higher-level phonological categories or rules (Edwards et al., 1999; Munson et al., 2005a, 2005b). Instead, aligning with the intervention terminology discussed in Wren et al. (2018), we propose referring to these as general contrastive (cognitive-linguistic) approaches and recommend avoiding the term “phonological” interventions. Furthermore, we recommend limiting contrast-based interventions to children who demonstrate stimulability in an imitation context (Miccio et al., 1999). Even the DEAP test manual (see section on diagnostic screening; Dodd et al., 2002) implicitly assumes that contrast-based interventions are suitable for stimulable errors.

Stimulability indicates the presence of underlying structural and functional integrity (Pollock and Rees, 1972; Powell and Miccio, 1996; Turton, 1973) and that a child can produce the required speech motor movements for target sounds when provided with appropriate scaffolding, such as visual, auditory, or tactile cues. However, the absence of stimulability does not necessarily mean that these motoric movements are beyond the child’s control. Rather, poor stimulability may stem from deficits in sensory input, difficulties in linguistic conceptualization, or other interfering factors such as attention (Powell and Miccio, 1996). Notably, stimulability is a more reliable predictor of intervention progress than traditional IPA transcriptions (Glaspey and Stoel-Gammon, 2007; MacLeod and Glaspey, 2014; Meyer and Munson, 2021). For children with limited or no stimulability, we advocate for a motor-based intervention approach, ideally incorporating biofeedback to enhance clinical outcomes (McAllister et al., 2022; To et al., 2022).

## 7 Conclusion

For over six decades, the influence of Natural Phonology (Stampe, 1969) has been deeply entrenched in Speech-Language Pathology, shaping the foundations of clinical practice, research, and education. Its principles have inspired generations of clinicians and academics, whose textbooks and assessment tools have been reprinted, reiterated, and reinforced without significant integration of speech motor development research or instrumental phonetics into diagnosis, intervention, or prognosis—with few notable exceptions (e.g., Edwards et al., 1999; Kent, 2024; Munson et al., 2005b). Dodd’s MDD (Dodd, 2014) has persisted largely unchanged, continuing to rely on outdated descriptive-linguistic classifications despite growing empirical and instrumental evidence that directly contradicts its core assumptions. Therefore, while such models may function as heuristics, it is premature to assume there is compelling evidence supporting their validity and reliability for widespread adoption (e.g., as proposed by Cleland et al., 2025; also see Csercsics et al., 2024; Stringer et al., 2024; Ttofari Eecen et al., 2019). Despite their failure to account for objectively verified, instrumentally derived speech data, the MDD (Dodd, 2014), along with other IPA transcription-based classification systems, including descriptive-linguistic models, psycholinguistic processing frameworks (e.g., Waring and Knight, 2013) and Shriberg et al.’s (2010) SDCS continue to be widely used in clinical practice. Why? Over three decades ago, Shriberg and Lof (1991) predicted that computers and instrumentation would revolutionize speech assessment by improving accuracy, reliability, and objectivity. Yet, nearly 30 years later, Munson et al. (2010) observed that this transformation had still not occurred. And now, 45 years later, in 2025, we find ourselves asking the same question: Why are we still relying on outdated, perception-based methods to classify speech development? Whether we blame technological inaccessibility, inadequate clinical training, or a failure in education to challenge outdated perspectives, one truth remains—in an era of Artificial Intelligence and cutting-edge instrumentation, persisting with outdated phonological classification models is a conscious choice, not an inevitability. The 1970s marked a pivotal shift in Speech-Language Pathology, as the term “functional articulation disorder” faded in favor of “developmental phonological disorders”—a transformation described by Shriberg and Kwiatkowski (1982) as a paradigm shift in the field. Today, we call for a new paradigm shift, urging recognition that underlying speech representations/plans are gesturally/motorically-informed (e.g., as defined by Articulatory Phonology). We emphasize that speech errors possess nuanced phonetic characteristics, offering valuable insights into speech maturation, assessment, and intervention strategies. Continued reliance on terms such as “phonological delay” and “phonological disorder” may incorrectly attribute causation to cognitive-linguistic deficits, thereby overlooking crucial motoric factors (Namasivayam et al., 2025). Persistent use of these outdated terms perpetuates classification models that no longer withstand empirical scrutiny. The evidence is here. The data is clear. The question is no longer whether we should shift, but why we have not already.
