# Supplementary material for: Reevaluating the classification of pediatric speech sound disorders: a ground truthing perspective
Source: Front Hum Neurosci. 2025 Dec 11;19:1700505. doi: 10.3389/fnhum.2025.1700505 (PMC12738827; doi:10.3389/fnhum.2025.1700505)
Supplement: Supplementary file 1 [file Data_Sheet_1.docx]

Supplementary Material

# Appendix A

*Case Example: Client A. Age: 5;7*

**Sample Client Profile Across Different Levels of Description**

*Tables describe the speech difficulties of one and the same child at different levels.*

**IPA Transcription of Error Patterns**

Selected sample of speech sound error patterns (IPA transcribed) observed on the Diagnostic Evaluation of Articulation and Phonology (DEAP; Dodd et al., 2002).

| **Pattern of Error** | **Word Target** | **Client’s Error**  **Transcription** |
| --- | --- | --- |
| Final Consonant Deletion | Pig | /pI/ |
|  | Teeth | /ti/ |
| Vowel Distortion + Final Consonant Deletion | Knife | /ne/ |
| Cluster reduction +vowel distortion | School | /sʊ/ |
| Stopping | Van | /bæn/ |
| Gliding + final consonant deletion | Orange | /owIn/ |
| Cluster reduction + final consonant deletion | Snake | /se/ |
| Syllable deletion + cluster substitution + gliding | Umbrella | /ʌfwɛjə/ |
| Cluster reduction + fronting | Swing | /fIn/ |
| Syllable deletion+ vowel distortion+ backing | Tomato | /əmego/ |

**Speech Motor Skill Limitations (Motor Speech Checklist: Namasivayam et al 2025)**

The following speech motor issues were noted during administration of 40 probe words to assess speech motor skills in children (Namasivayam et al., 2021). The total motor error score was 18, and the child had difficulty on 17 of the 18 items.

| **Jaw Control** | | | | | | | | |
| --- | --- | --- | --- | --- | --- | --- | --- | --- |
| Jaw 13.1 | Inadequate jaw opening (over extension/ too restricted) | | | | | Yes | | |
| Jaw 13.2 | Inability to grade jaw for mid heights vowels /I, e, ε, e, o, ∧/ | | | | | Yes | | |
| Jaw 13.3 | Decreased jaw stability/ decreased mid-line jaw control (lateral/anterior) sliding | | | | | Yes | | |
| **Labial-Facial Control** | | | | | | | | |
| LF 14.1 | Inadequate bilabial contact for /p/m/b/ with any vowel | | | | | No | | |
| LF 14.2 | Non-independent bilabial movement from jaw for /p/b/m/ to high vowels (/i, I, e, u/) and contribution of upper/lower lip is unequal (beep) | | | | | Yes | | |
| LF 14.3 | Lower lip movement for /f/, is not independent from jaw e.g. “feet” | | | | | Yes | | |
| LF 14.4 | Inadequate lip rounding for /o/ and /u/ (no jaw help) e.g. “no”, “boot” | | | | | Yes | | |
| LF 14.5 | Inadequate lip retraction for /i/, /e/ (symmetrical with no “fixing” at lip corners) | | | | | Yes | | |
| **Integration of Jaw + Lip (2-plane movement)** | | | | | | | | |
| Integrate 15.1 | | | | Jaw and Lips - Inadequate jaw range with lip rounding for /au/ e.g. “down” | | | Yes | |
| Integrate 15.2 | | | | Jaw and Lips - Inadequate jaw range with lip retraction for /ai/ or across 2 syllables e.g. “bite”, “mommy” | | | Yes | |
| **Lingual Control** | | | | | | | | |
| Lingual 16.1 | | | Non-independent tongue tip elevation from jaw for /t,d,n/ e.g. “two”, “no” | | | | Yes | |
| Lingual 16.2 | | | Inaccurate posterior movement (velar /k/ and /g/ e.g. “cookie”, “go”) | | | | Yes | |
| **Multi-Plane Movements** | | | | | | | | |
| MP 17.1 | | Inability to alternate lip retraction with lip rounding e.g. “yoyo” | | | | | | Yes |
| MP 17.2 | | Inability to produce multi-syllabic words with change of place and plane of movement e.g. “ladybug”, “doubleyou” | | | | | | Yes |
| **General Speech Production Characteristics** | | | | | | | | |
| Limited Variety | | | | | Limited variety of speech motor movements (e.g. uses jaw as primary articulator, ie. jaw-supported speech). | | | Yes |
| Limited Vowel | | | | | Child presents with limited vowel repertoire and/or vowel distortions or a limited consonant repertoire and/or consonant distortions. | | | Yes |
| Limited Shapes | | | | | Child presents with limited syllable and word shapes | | | Yes |
| Length Complexity | | | | | Child has difficulty maintaining sound and syllable integrity with increased length and complexity of utterance. | | | Yes |

**Dimensional Approach to Speech Sound Disorders**

| **Area** | **Potential Assessments** | **Client Level Factors** | **Intervention Level** |
| --- | --- | --- | --- |
| **Speech Motor Skills and**  **Speech Differentiation between articulators** | Verbal Motor Production Assessment of Children-R (Harden & Namasivayam, 2021)  The Kaufman Speech Praxis Test (KSPT; Kaufman, 1995)  Probe Word List to Assess Speech Motor Skills in Children (Namasivayam et al., 2021). | Client mostly produces CV, CVCV, and VV syllable shapes; difficulty with CVC, VC, and CCVC. | Client benefits from temporal cues, somatosensory cues, mass/blocked practice, and knowledge of performance feedback to improve motor learning for shaping and developing motor speech movements. |
| Language Skills | Comprehensive Assessment of Spoken Language–Second Edition (CASL-2; Carrow-Woolfolk, 2017)  Preschool Language Scales–Fifth Edition (PLS-5; Zimmerman et al., 2011) | Child demonstrates age appropriate receptive language skills; expressive language limited by reduced speech intelligibility. | Therapist uses strength of receptive language ability to explain speech motor expectations/cues and provides specific, descriptive feedback to improve expressive language production. |
| Literacy Skills | Test of Preschool Early Literacy (TOPEL; Lonigan et al., 2007) | Child demonstrates age appropriate letter recognition and identification. Sound symbol relationships are impacted by reduced articulatory accuracy. | The clinician provides multisensory instruction to pair symbols with sounds correctly (e.g., the letter “g” corresponds to /g/). For example, the clinician shows the letter “G” on a whiteboard while giving tactile input to the mylohyoid muscle to cue the movement for /g/. The clinician leverages the client’s knowledge of letters to build motor speech movements. |
| Functional Communication Skills | The Functional Communication Profile - Revised (FCP-R; Kleiman, 2003)  Focus on the Outcomes of Communication Under Six (FOCUS; Thomas‐Stonell et al., 2013) | Uses language for a variety of purposes; however, limitations in speech intelligibility impacts comprehension from the listener often resulting in communication breakdowns. | Use meaningful, functional words that can serve multiple pragmatic purposes (“power words”). For example, pair the speech-motor target “go” (posterior lingual control plus lip rounding) with the meaning of “go” to request an action, respond to a question, or direct another person. |
| Self-Regulation / Social-Emotional Skills | The Child Sensory Profile -2 (SP-2; Dunn, 2014) administered by an occupational therapist | The client is easily distracted by the environment and shows limited task attention without sufficient motivation. | The clinician gradually increases repetition thresholds, uses highly motivating/preferred activities, and incorporates games into therapy. The client is given access to sensory equipment (e.g., trampoline, swings) before/during speech therapy for self-regulation. |
| Executive Functioning/ Cognitive Flexibility/ Working Memory | Behavior Rating Inventory of Executive Function- 2nd Edition (BRIEF-2; Gioia et al., 2015).  Developmental [Neuropsychological Assessment](https://www.sciencedirect.com/topics/psychology/neuropsychological-assessment) (NEPSY; [Korkman, Kirk, and Kemp, 2007](https://www.sciencedirect.com/science/article/pii/S1750946716300320?casa_token=3LUdJ6EEQccAAAAA:H9-JaG2bsGvwP_l-UU2A8-Dow5p0D6vUJvglGQ-ivSSd6M-fKJRqqmz940bs2aTlM0IVaojimjY#bib0125)) | Child demonstrates reduced motor learning. Retention of motor speech targets requires significant attention, repetition and prompting. | Clinician has developed a strong home program for daily carryover. Temporal cueing used to practice direct and delayed imitation of targets to support speech motor target retention. |
| Sensory Perceptual and Discrimination Abilities | Auditory Processing Abilities Test (APAT; Ross-Swain and Long, 2004)  Two-point discrimination and oral stereognosis tests (Boliek et al., 2007; Jacobs et al., 2002) | The child shows reduced attention to visual and auditory input, making it difficult to focus on models based on speech sounds alone (e.g., “say this sound”).  The child demonstrates adequate oral sensory discrimination. | The clinician uses a multisensory approach by showing the client which muscles to use and how they should move (e.g., tactile input on the lips for closure and a mirror for visual feedback). The client cannot rely on auditory information alone to match sound-based models. |

***Note : Additional assessments may be added as required (e.g., phonological awareness, vocabulary, speech perception/discrimination etc)***

**References:**

Boliek, C. A., Rieger, J. M., Li, S. Y. Y., Mohamed, Z., Kickham, J., & Amundsen, K. (2007). Establishing a reliable protocol to measure tongue sensation. *Journal of Oral Rehabilitation*, *34*(6), 433-441.

Carrow-Woolfolk E. (2017). *Comprehensive Assessment of Spoken Language, Second Edition (CASL-2)* [Manual]. Torrance, CA: Western Psychological Services.

Dunn, W. (2014). Sensory Profile 2: User's manual. Bloomington, MN: Pearson.

Gioia, G. A., Isquith, P. K., Guy, S. C., & Kenworthy, L. (2015). *Behavior Rating Inventory of Executive Function, Second Edition (BRIEF-2).* PAR, Inc.

Hayden, D., & Namasivayam, A. K. (2021). *Verbal Motor Production Assessment for Children-Revised (VMPAC-R)* [Mobile application software]. <https://vmpac-r.com/>

Jacobs, R., Wu, C. H., Van Loven, K., Desnyder, M., Kolenaar, B., & Van Steenberghed, D. (2002). Methodology of oral sensory tests. *Journal of oral rehabilitation*, *29*(8), 720-730.

Kaufman, N. R. (1995). *Kaufman Speech Praxis Test for Children (KSPT).* Wayne State University Press.

Korkman, M., Kirk, U., & Kemp, S. (2012). *NEPSY-II: administration manual*. NCS Pearson.

Kleiman, E. M. (2003). *Functional Communication Profile–Revised (FCP-R)*. LinguiSystems.

Lonigan, C. J., Wagner, R. K., Torgesen, J. K., & Rashotte, C. A. (2007). *Test of Preschool Early Literacy (TOPEL).* Pro-Ed.

Namasivayam, A. K., Huynh, A., Bali, R., Granata, F., Law, V., Rampersaud, D., ... & Hayden, D. (2021). Development and validation of a probe word list to assess speech motor skills in children. *American Journal of Speech-Language Pathology*, *30*(2), 622-648. <https://pubs.asha.org/doi/full/10.1044/2020_AJSLP-20-00139>

Namasivayam, A. K., Li-Han, L. Y., Moore, J., Wong, W., & van Lieshout, P. The Articulatory Basis of Phonological Error Patterns in Childhood Speech Sound Disorders. *Frontiers in Human Neuroscience*, *19*, 1635096. <https://doi.org/10.3389/fnhum.2025.1635096>

Thomas‐Stonell, N., Oddson, B., Robertson, B., & Rosenbaum, P. (2013). Validation of the Focus on the Outcomes of Communication under Six outcome measure. *Developmental Medicine & Child Neurology*, *55*(6), 546-552.

Ross-Swain, D., & Long, N. (2004). *APAT: Auditory Processing Abilities Test*. Academic Therapy Publications.

Zimmerman, I. L., Steiner, V. G., & Pond, R. E. (2011). *Preschool Language Scales, Fifth Edition*. The Psychological Corporation.
