# Supplementary material for: Reevaluating the classification of pediatric speech sound disorders: a ground truthing perspective
Source: Front Hum Neurosci. 2025 Dec 11;19:1700505. doi: 10.3389/fnhum.2025.1700505 (PMC12738827; doi:10.3389/fnhum.2025.1700505)
Supplement: Supplementary file 2 [file Data_Sheet_2.docx]

Supplementary Material

# Appendix B

**Recommendations for how the field of Speech-Language Pathology can overcome the barriers of technological education**

Strengthening the technological base of clinical education is challenging because the graduate curriculum is densely packed with coursework. We take acoustics as an example of expanded technological preparation because it has several advantages in clinical education and application, including the following:

(a) Instruction in basic acoustics already is included in most undergraduate or graduate curricula in speech-language pathology. Textbooks on speech science usually include one or more chapters on acoustics, providing an opportunity for a seamless instructional path from undergraduate to graduate coursework.

(b) Important concepts can be taught to students who do not have a strong background in physics or mathematics (Arai, 2022). An advanced degree in the physical sciences is not required to conduct acoustic analysis of speech.

(c) Tutorials are available to demonstrate the clinical utility of acoustic methods for the analysis of speech and voice (Bunta & Gósy, 2022; Neel, 2010; Shahin et al., 2019; Walden, 2024),

(d) Acoustic databases are being established for diverse speaker groups, including individuals with speech and language disorders across the lifespan.

(e) Software for acoustic analysis is available at little or no cost and does not require specialized hardware other than a computer. A prime example is the freeware Praat (Boersma & Weenink, 2025), which is widely used by phoneticians, speech scientists, and others concerned with speech communication.

(f) Acoustics is applicable to multiple aspects of speech, including articulation, resonance, voice, prosody, dialect, and emotion.

(g) Acoustic analysis is incorporated in speech technologies that have either demonstrated or potential value in clinical services.

Taken together, these factors offer a tractable solution to enhance technological training in speech-language pathology. Pedagogical resources are readily available to support both lectures and laboratory activities.

**References**

Arai, T. (2022). Education in basic acoustics for acoustic phonetics and speech science. The Journal of the Acoustical Society of America, 152(5), 2746-2757.

Boersma, P. & Weenink, D. (2025). Praat (Version 6.4.27) [Computer software]. Retrieved from http://www.fon.hum.uva.nl/praat/

Bunta, F., & Gósy, M. (2022). Expanding the clinical toolset: acoustic analyses for speech-language pathologists and audiologists in the 21st century. Perspectives of the ASHA Special Interest Groups, 7(6), 2146-2157. https://doi.org/10.1044/2022_persp-22-00090

Neel, A. T. (2010). Using acoustic phonetics in clinical practice. Perspectives on Speech Science and Orofacial Disorders, 20(1), 14-24. https://doi.org/10.1044/ssod20.1.14

Shahin, M., Zafar, U., & Ahmed, B. (2019). The automatic detection of speech disorders in children: Challenges, opportunities, and preliminary results. IEEE Journal of Selected Topics in Signal Processing, 14(2), 400-412. doi:10.1109/JSTSP.2019.2959393

Walden, P. R. (2024). Learning acoustic assessment of voice: an action research-based SoTL Project. Journal of Voice, 38(4), 966-e1. https://doi.org/10.1016/j.jvoice.2021.11.015
